# Is patient empowerment the key to promote adherence? A systematic review of the relationship between self-efficacy, health locus of control and medication adherence

**DOI:** 10.1371/journal.pone.0186458

**Published:** 2017-10-17

**Authors:** Lilla Náfrádi, Kent Nakamoto, Peter J. Schulz

**Affiliations:** Institute of Communication and Health, Università della Svizzera italiana, Lugano, Switzerland; University of the West Indies Faculty of Medical Sciences Mona, JAMAICA

## Abstract

**Background:**

Current health policies emphasize the need for an equitable doctor-patient relationship, and this requires a certain level of patient empowerment. However, a systematic review of the empirical evidence on how empowerment affects medication adherence—the extent to which patients follow the physician’s prescription of medication intake—is still missing. The goal of this systematic review is to sum up current state-of-the-art knowledge concerning the relationship between patient empowerment and medication adherence across medical conditions. As our conceptualization defines health locus of control and self-efficacy as being crucial components of empowerment, we explored the relationship between these two constructs and medication adherence.

**Methods:**

Relevant studies were retrieved through a comprehensive search of Medline and PsychINFO databases (1967 to 2017). In total, 4903 publications were identified. After applying inclusion and exclusion criteria and quality assessment, 154 articles were deemed relevant. Peer-reviewed articles, written in English, addressing the relationship between empowerment (predictor) and medication adherence (outcome) were included.

**Findings:**

High levels of self-efficacy and Internal Health Locus of Control are consistently found to promote medication adherence. External control dimensions were found to have mainly negative (Chance and God attributed control beliefs) or ambiguous (Powerful others attributed control beliefs) links to adherence, except for Doctor Health Locus of Control which had a positive association with medication adherence. To fully capture how health locus of control dimensions influence medication adherence, the interaction between the sub-dimensions and the attitudinal symmetry between the doctor and patient, regarding the patient’s control over the disease management, can provide promising new alternatives.

**Discussion:**

The beneficial effect of patients’ high internal and concurrent physician-attributed control beliefs suggests that a so-called “joint empowerment” approach can be suitable in order to foster medication adherence, enabling us to address the question of control as a versatile component in the doctor-patient relationship.

## Introduction

Medication non-adherence—defined as the extent to which patients take medication in ways other than those prescribed by their health care providers—is a serious obstacle to chronic disease care, with a 50% average prevalence across conditions [1,2]. Non-adherence has numerous patient, physician, medication and health care system related factors [1,2]. Several characteristics of the patients, such as health literacy and medication beliefs influence adherence [1–4]. Empowerment [5]—as an activating force that motivates some people to take their health behavior and management of illnesses into their own hands—is also one of the patient-related factors. Empowerment can be conceived as a personal disposition, referring to the patient’s control and power in the medical context [6] or as a relational concept, emphasizing the existing equity in the physician-patient relationship [7]. A collaborative doctor-patient relationship can improve patient empowerment, i.e. the lack of concordance between doctors and patients can lead to paternalism, and negotiated care can bring power balance into the medical relationship [8]. As a matter of fact, the physicians by facilitating patient engagement in the communication process can foster patient empowerment and better patient outcomes [9].

Patient empowerment has been associated with positive health and clinical outcomes since the concept made its mark in health care literature [5,10,11]. The outcomes considered [12] include improved disease management [13–17], effective use of health services [13–15,18], improved health status [19–21], and medication adherence [22,23]. The association between empowerment and positive health behavior and clinical outcomes generally rests on the assumption that patient autonomous activity is beneficial for their health condition. However, in the case of medication adherence, this assumption might not always hold true. Highly empowered patients might believe that they can make treatment decisions more or less by themselves overruling the physician’s prescription. Indeed, intelligent non-adherence is becoming a common term in the adherence literature, referring to patients´ intentional non-adherence based on rational reasons, such as misdiagnosis or side-effects [24]. This definition presupposes that non-adherence in case of these patients is the “right” choice which leads to beneficial health outcomes, i.e. the patients are adequately health literate (having necessary knowledge and decision-making skills about the medical treatment [25,26] to judge whether adhere or not to the prescribed medication regimen. Similarly, Bader et al. (2006) defined critical adherence as the patients’ freedom to elect to interrupt or forgo a therapy, based on an autonomous evaluation [27]. On the one hand, these forms of non-adherence are often viewed as necessary, since adherence is favorable only if the medication is beneficial. On the other hand, if such a high level of patient autonomy is not accompanied by an equally high level of health literacy, the patient might be inclined to non-adherence in a presumptuous manner putting his/her health into jeopardy [25]. Indeed, a recent review [28] investigating the consequences of increasing patient empowerment reported that a high level of patient empowerment had a controversial relationship with adherence. Some aspects of patient empowerment (such as information search and knowledge) promote adherence, while others (i.e. decision participation) reduce therapy adherence [28]. These challenges call for all concerned to review the empirical knowledge available on the relationship between empowerment and adherence.

A multidimensional conceptualization proposed originally in management literature [29,30], and adapted to the health context by Schulz and Nakamoto [31], perceives empowerment as a motivational construct, holding that patients participate as autonomous actors in health care decisions and consequentially take increased responsibility for such decisions [31]). This concept has four components: 1. Meaningfulness (refers to the value of activities), 2. Competence (belief in one’s own capabilities), 3. Impact (belief in making a difference), and 4. Self-determination (refers to the extent to which a choice is characterized by autonomous initiation). Based on this conceptualization, in the present review, patient empowerment is operationalized as patients’ perceptions of their own capacity for disease management and their beliefs about how much control they have over their own health outcomes. This definition leads to two main constructs constituting empowerment, which have been widely studied in medication adherence literature: self-efficacy and health locus of control.

Self-efficacy is strongly related to the Competence dimension of the empowerment concept. Self-efficacy draws on social-cognitive theory and can be defined as the individual’s belief in his/her own ability to implement a specific behavior or a set of behaviors [32]. It can refer to general [33] or context-specific perceptions of one´s own capacity to mobilize resources and motivation to deal with certain situations and challenges [33]. General self-efficacy refers to a stable sense of personal competence across situations [34,35]. It has been shown that a high sense of efficacy can be associated with better health outcomes, greater achievement and better social integration [32,36]. Moreover, self-efficacy was found to be the cornerstone of medication adherence in chronic mental illness [37] and the most prominent factor of adherence across conditions within the socio-cognitive and self-regulation theories [38].

Health locus of control (HLOC) considers whether an individual’s source of reinforcement for health-related behavior is internal or external. The former relates to a high, the latter to a low level of Impact and Self-determination. The operationalization of the construct has since gone through significant changes. A first approach by Rotter (1966) considered Internal (ILOC) and External locus of control (ELOC) as two endpoints of a one-dimensional continuum [39]. ILOC means that the individual’s sense of control over their health is directly related to their own actions, while ELOC refers to the perception that one’s health is determined by external factors. A later conceptualization, the Multidimensional Health Locus of Control (MHLOC), kept internality (Internal HLOC), but divided external locus of control (External HLOC) into specified sub-dimensions: Powerful Others, Doctor, Chance and God HLOC [40,41]. HLOC has been widely used as a predictor of health behavior. Generally, it is assumed that people with high Internal HLOC are more likely to behave in healthier ways than those who do not believe that they have control over their health. However, an extremely high level of Internal HLOC has been proposed to be problematic [42]. By contrast, someone with high scores on the Chance HLOC subscale, believing that it is fate or chance that determines his/her health status, is probably less likely to implement recommended health behavior. Powerful Others HLOC is generally not assumed to encourage health behavior, except if others are supportive of engaging in healthy behavior [43–45]. Wallston (2005) suggested incorporating other variables such as self-efficacy as potential moderators when investigating how HLOC influences health behavior [45]. This suggestion goes hand in hand with our empowerment concept as it incorporates self-efficacy and health locus of control.

The role of patient empowerment in influencing medication adherence is a cardinal question with major practical implications across all clinical and policy levels. Therefore, the present systematic review seeks to answer the question, whether high level of patient empowerment is associated with greater medication adherence. Thus, the goal is to assess the relevant empirical findings about the relationship between patient empowerment and medication adherence across medical conditions. Medication non-adherence was defined as the extent to which the patients do not agree with/follow the physician’s recommendations regarding taking the prescribed medication, measured by self-report, objective measures or mixed indicators. As empowerment is regarded as consisting of HLOC and self-efficacy, we will address their influence on medication adherence in two separate steps.

## Materials and methods

### Inclusion and exclusion criteria

The studies had to meet the following criteria in order to be included in the present systematic review: (1) peer-reviewed articles, (2) written in English, (3) studies having observational or experimental design, (4) addressing the relationship between medication adherence and at least one aspect of empowerment, (5) empowerment should be considered either as an independent variable or as a mediator, (6) medication adherence had to be assessed as an outcome variable and (7) studies including an adult sample. We excluded papers which were not available in English, included a sample of youth, or focused on over the counter medications. Moreover, qualitative studies, commentaries, essays, study protocols, literature reviews, conceptual papers and conference abstracts were also excluded.

### Search strategy

In order to select a proper search strategy, a set of keywords was identified based on a prior scoping search identifying the relevant academic jargon. Therefore, we initially searched several databases including PROSPERO, COCHRANE library, Medline and PsychInfo for review articles related to medication adherence and/or empowerment. Subsequently, a Thesaurus and a PubMesh search was conducted in order to complete the list. In the main search, key terms referring to empowerment and its related constructs were combined with either words related to medication adherence or misuse. In order to include as many variants of the key terms as possible, a wildcard search was used where this was deemed necessary. Keywords were connected via the following Boolean operators: ‘Empowerment’ AND ‘Medication’ AND (‘Adherence’ OR ‘Misuse’) (see: Table 1).

**Table 1:** The Boolean search strategy applied in the present systematic review.

Empowerment	Medication	Adherence	Misuse
empower* OR self-efficacy OR self-determin* OR locus of control OR perceived autonomy OR perception of autonomy OR overconfidence	medic* OR drug OR prescrip*	adherence OR non-adherence OR compliance OR non-compliance OR concordance OR non-concordance OR autonomous regulation of medication taking	self-medication OR misuse OR abuse

In order to identify relevant articles, two online databases were searched, one with a medical orientation (Medline) and the other with a focus on social sciences (PsychInfo). The search included the literature published since 1966 to May 2017 in Medline and the publications in the time period between 1967 to May 2017 in PsychInfo.

### Data analysis and synthesis

The review was prepared in accordance with the PRISMA statement [46] (S1 Table). The original search was carried out by one of the authors (L.N.), and yielded 4903 hits, which were then screened on the basis of the titles and abstracts using three coders for their relevance, applying the above-mentioned inclusion criteria. The interrater agreement was adequate (Cohen’s kappa = 0.95) regarding which publications were deemed relevant and included for full text assessment. A 13-item checklist developed by Wallace et al. (2006) was applied to assess the quality of the included studies. The checklist contains specific questions in order to assess whether the authors provide a clearly defined research question and appropriately describe the theoretical background. Further questions probe the rigor of study design, data collection, data analysis and the solidness of the derived conclusions [47]. The scores ranged from 0 to 13. The interrater agreement regarding the quality of the papers was 79%. The interrater agreement was calculated based on 10% of the hits, which is the recommended ratio of articles to be coded by independent coders according to rule of thumb [48]. Relevant data, such as information regarding the sample, methodology, the included measurement tools and outcomes were extracted based on a previously developed coding scheme. In the case of the data extraction, the interrater agreement was 85.6%. The coders of the abstract screening, data extraction and quality assessment included one of the authors (L.N.) and research assistants with a health communication background. Meta-analysis was not considered to be appropriate as the included studies demonstrated large heterogeneity in terms of the conceptualization and operationalization of empowerment as well as medication adherence [49].

## Results

**Fig 1:**
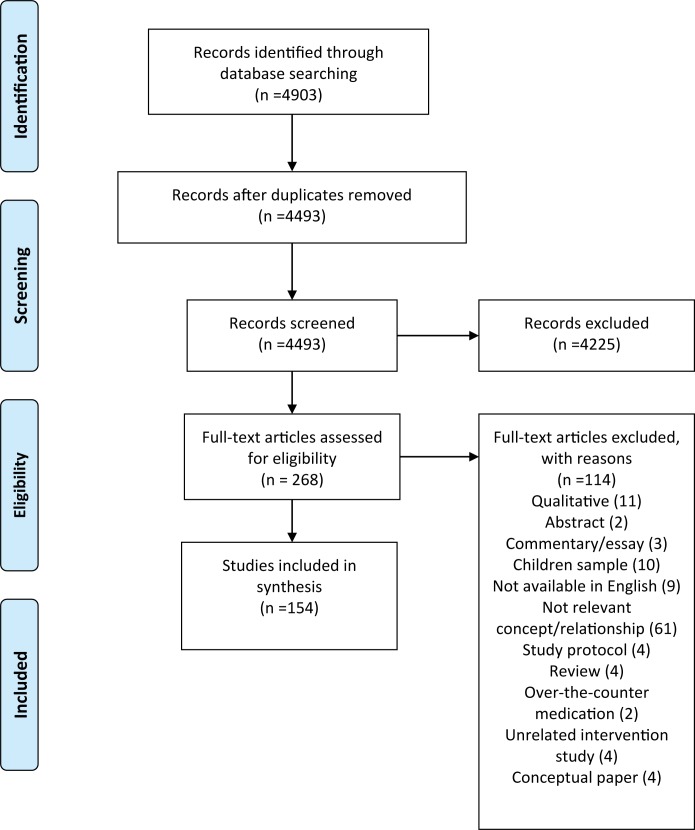
Flow diagram of the study selection process, adapted from Moher et al. (2009).

154 articles were deemed to be fit for inclusion based on the full text analysis. None of the studies were judged to have a quality so poor that they had to be excluded. The average quality score was 11.93 out of 13 (SD = 1.6). If the articles reported more than one outcome about the relationship between the empowerment-related constructs and adherence, each result was included separately in the review. Fig 1 provides an overview of the selection process.

### Overview of the results

The vast majority of the studies were conducted in North America (n = 107). Other studies took place in Europe (n = 25), Asia (n = 11), Eurasia (n = 1), Australia) (n = 5), South America (n = 4) and Africa (n = 1).

The studies involved patients diagnosed with various disease groups: HIV/AIDS (n = 52), heart and vascular disorders (n = 18), respiratory diseases (n = 15), renal (n = 12) and of the endocrine system (n = 11). Some studies involved participants suffering from diseases of the brain and nervous system (n = 8), musculoskeletal (n = 6) and digestive system (n = 4), cancer (n = 4) and other conditions (n = 17). A small number of studies concerned patients diagnosed with psychological conditions (n = 7). S2 Table provides an overview of the sample characteristics.

We identified numerous scales measuring a given dimension of empowerment based on our operational definition. As far as locus of control is concerned, most of the studies applied either a one dimensional (RIELCS) [39] or a multidimensional measurement (MHLOC Scale) [40] of the concept. Our search revealed studies using several types of self-efficacy beliefs: medication adherence, disease-management, general, coping self-efficacy, etc. Only a minority of the included articles focused on other empowerment-related constructs, such as competence or self-regulation.

Concerning the operationalization of medication adherence, we can distinguish three types: **objective measures** (using pill count, patients’ medical outcomes or pharmacy records as a proxy of adherence), **subjective measures** i.e. where patients provided self-reported questionnaires or structured interviews, and lastly **mixed measures** which apply subjective and objective adherence measures simultaneously. As there is no agreement on a single method which performs well across all criteria [50], we decided to include all of the studies regardless of the adherence measure applied. See S3 Table for an overview of the included studies and S4 Table for their quality assessment score.

### The relationship between self-efficacy and medication adherence

The identified studies addressing the relationship between adherence and self-efficacy focused mainly on medication adherence, disease-management and general self-efficacy (See: Table 2).

**Table 2:** The relationship found between different types of self-efficacy and medication adherence (N = 92).

Relationship with medication adherence		Positive relationship	Negative relationship	No relationship	Mediator	Mixed results	All
Types of self-efficacy	Medication adherence	89% (n = 59)	-	6% (n = 4)	5% (n = 3)	-	100% (n = 66)
	Disease management	80% (n = 16)	-	15% (n = 3)	-	5% (n = 1)	100% (n = 20)
	General	66,6% (n = 4)	-	33,3% (n = 2)	-		100% (n = 6)

**Medication adherence self-efficacy** refers to a belief in the patient’s capacity to follow a prescribed medical regimen in challenging situations [51]. Most of the articles (59 out of 66 studies) found a positive link between medication adherence self-efficacy and adherence [52–100], and among these 9 studies also reported that self-efficacy had a mediator role [101–110]. Only three studies considered adherence-self-efficacy solely as a mediator [111–113] and four reported no relationship [114–117].

**Disease management self-efficacy** refers to the patients’ beliefs in their capacity to manage disease in general [118,119) or condition-specific self-management behavior [120,121]. The studies investigating the relationship between disease management self-efficacy and medication adherence applied specific scales developed to measure efficacy beliefs about implementing tasks related to managing a given condition. The majority of the studies (16 out of 20) reported a positive association [118,122–136], while only 3 reported no relationship [119,137,138] and 1 suggested mixed results [139].

**General self-efficacy** refers to one’s perceived competence across a wide array of life domains (36). General self-efficacy had, in most of the cases (4 studies out of 6), a positive association with adherence [140–143], while 2 studies did not find any relationship [144,145]. Some studies simultaneously applied general as well as disease or drug specific self-efficacy measures and investigated which one was more likely to predict self-reported adherence to medication. Two of the studies reported that disease specific self-efficacy was a more important predictor of adherence than generic self-efficacy [142,146]; the third study reported no relationship [145].

Studies focusing on **other domain-specific self-efficacy measures** reported a positive link between adherence and self-efficacy in patient-physician interactions [147,148], coping self-efficacy [149], bi-cultural self-efficacy [150], self-efficacy for managing negative mood, adhering to medication, symptoms and fatigue management, communicating with health care providers and getting support from others [151]. Self-efficacy when disclosing drug use to providers and for safer drug use were not as effective predictors of self-reported adherence as adherence self-efficacy [52]. Self-efficacy scores for physical function were seen to have a negative link to adherence[145].

**Concerning the different adherence measures**, a positive association between self-efficacy and adherence was reported in a greater proportion in studies using subjective (87%) or mixed measures (79%), compared to studies applying objective adherence measures (66.6%).

### The relationship between HLOC and medication adherence

HLOC was predominantly measured using two instruments: the one-dimensional RIELCS measure [39] and the MHLOC [41,152,153], which is a multidimensional instrument.

First, we considered the relationship between the RIELCS and medication adherence. Of the 9 studies, in four cases (45%) ILOC was found to be more beneficial for medication adherence than ELOC [154–157]. By contrast, two studies (22%) found that ELOC was related to better medication adherence than ILOC [158,159]. Finally, in three (33%) studies, ILOC and ELOC orientation did not distinguish between compliant and non-compliant patients [160–162].

The results which relate to **MHLOC** dramatically differ in terms of its sub-dimensions: Internal HLOC and External HLOC. Many studies (10 out of 26 studies) reported a positive association between **Internal HLOC** and adherence [81,101,163–170]; only a single study found a negative relationship [171]. Another study reported that a high level of Internal HLOC promotes critical adherence or non-adherence, i.e. how patients decided to follow or forgo a therapy based on autonomous evaluation [27]. There was a high number of null findings, i.e. 15 studies reported no relationship between IHLOC and adherence [160,172–185].

Examining the MHLOC External sub-dimensions respectively (i.e. Chance, God, Powerful others and Doctor HLOC) allowed us to explore and compare their variable associations with adherence. A large enough proportion of the studies (5 out of 18) found a negative link between **Chance HLOC** and adherence [170,183,186–188], while only one study reported a positive relationship (189). Twelve studies reported null findings [172,174–177,179,180,182,183,185,190,191]. **God HLOC** had a negative association with medication adherence [172]. In the case of the **Powerful others HLOC**, the findings are ambiguous, as the number of studies reporting a negative relationship with adherence [169,181,183,192,193] is only slightly higher (5 studies) than the studies which found a positive link (4 studies) [171,189,194,195]. In addition, 11 studies reported no relationship [172,174–177,180,182–184,191,196]. **Doctor HLOC,** by contrast, seems to promote adherence, as studies unequivocally found that people characterized with high Doctor HLOC tend to comply better with their given medical regimen (3 studies) [169,172,194]. However, in this case 3 studies also found no association with adherence [174,183,197] (see: Table 3).

**Table 3:** The relationship between MHLOC and adherence (N = 71).

MHLOC dimension		Positive relationship	Negative relationship	No relationship	All
Internal HLOC		38% (n = 10)	4% (n = 1)	58% (n = 15)	100% (N = 26)
External HLOC	Powerful others	20% (n = 4)	25% (n = 5)	55% (n = 11)	100% (n = 20)
	Doctor	50% (n = 3)	-	50% (n = 3)	100% (n = 6)
	God	-	100% (n = 1)	-	100% (n = 1)
	Chance	5% (n = 1)	28% (n = 5)	67% (n = 12)	100% (n = 18)

The studies applying subjective adherence measures reported more association either in a positive or negative direction (58.5%) between the HLOC dimensions and adherence compared to the studies applying mixed (14.3%) or objective (40%) adherence measures.

HLOC can also be conceived as a **mediator** of the relationship between adherence and various other predictors, such as perceived necessity of the treatment [198], social support [199], self-efficacy and outcome expectancy [200], competence [175,201] and hostility [202]. Alternatively, MHLOC can also explain to what degree any identified medication non-adherence is deliberate [203].

### Health condition-specific patterns

Regarding the role of self-efficacy in predicting adherence across health conditions, in patients diagnosed with digestive system or musculoskeletal diseases, multiple sclerosis, epilepsy, headache and psychiatric conditions, all studies reported a positive association between adherence and self-efficacy. In HIV, respiratory diseases, cancer, heart and vascular disorders, diabetes and renal diseases, the majority of the studies reported a positive association between adherence and self-efficacy, but there were some null findings, too.

Concerning how HLOC affects medication adherence, the studies involving patients diagnosed with HIV, cancer, diabetes and digestive system diseases reported that Internal HLOC fosters, while External HLOC dimensions appear to hinder adherence. In other conditions, the relationship between adherence and HLOC was more ambigous, i.e. in heart and vascular disorders and mental conditions. Besides Internal HLOC, high levels of Doctor HLOC emerged as a significant predictor of greater adherence in two conditions: respiratory diseases [172,194] and renal disease [169,201].

Other important predictors of adherence were the patients’ autonomy preference regarding respiratory diseases [204], perceived autonomy among renal transplant patients [205], self-regulation in heart failure patients [206] and perceived competence [207], autonomous motivation [180,207] and treatment-related empowerment [208] in HIV. In patients diagnosed with diabetes, perceived competence [209], diabetes empowerment [22] and Diabetes HLOC [210] were predictors of adherence.

## Discussion

In the present study, the relationship between patient empowerment and medication adherence was reviewed, with special focus on the two constructs deemed to be the central facets of empowerment: HLOC and self-efficacy.

### An overview of the relationship between self-efficacy and adherence

Our results confirm that self-efficacy is a strong predictor of medication adherence. Positive association with adherence holds for of all types of self-efficacy: general, medication adherence, disease management and other domain specific measures. Our findings confirm and expand the findings of McCann et al.’s review [37], which highlighted the importance of self-efficacy in medication adherence in chronic mental illness, as we found that self-efficacy was an important predictor of adherence both in the case of mental and somatic diseases. On the one hand, the effect of self-efficacy in fostering adherence is so robust that it holds regardless of the type of self-efficacy applied and across all medical conditions. On the other hand, specific measures of both medication adherence self-efficacy and disease management self-efficacy show a positive association with adherence compared to general self-efficacy more consistently. Thus, future research might apply context-specific measures for predicting medication adherence, considering that the most consistent adherence self-efficacy seems to be associated with adherence. However, this strong link might arise from the operational overlap between these constructs, as medication adherence questionnaires sometimes measure barriers to medication adherence, such as certain aspects of adherence self-efficacy [50].

### The relationship between HLOC and adherence

The findings show that Internal HLOC predominantly has a positive link with medication adherence, while External HLOC dimensions have variable associations.

Studies applying the one-dimensional RIELCS [39] showed a trend suggesting that ILOC is more favorable for adherence than ELOC, but the difference was small. The characteristics of this measure might account for the contradicting findings, as the RIELCS does not distinguish the dimensions of ELOC, and this might lead to individual differences in the interpretation. Alternatively, treating ILOC and ELOC as opposite ends of one continuum may not capture its effect on adherence. Therefore, the MHLOC Scale [40] seems to be a more appropriate tool when examining the relationship between HLOC and adherence.

As far as MHLOC is concerned, a clear majority of the studies reported a positive relationship between Internal HLOC and adherence, which confirms that patients’ beliefs of being in control of their own health (a facet of high empowerment) is associated with greater adherence. However, the relationship between External HLOC and adherence is variable, as the sub-facets of HLOC show variable relationships with adherence. In accordance with our assumptions, Chance HLOC, which can signal low empowerment, showed a negative association with medication adherence. God HLOC also had a negative link to adherence, but only one study looked into this relationship [172]. The number of studies reporting a positive and a negative association between Powerful others HLOC and adherence were almost equal. The effect of Powerful others HLOC on adherence might depend on whether these others support or discourage the patients to adhere [43–45]. Indeed, Doctor HLOC seems to be the most beneficial element of External HLOC promoting adherence, especially when it was concurrent with high Internal HLOC [169]. The variable associations of different External HLOC dimensions with medication adherence indicate that the perceived control dimension of empowerment is far from homogenous. In particular, Doctor HLOC fosters adherence, highlighting how, in a medication management context, not only the belief in one’s own ability and control are beneficial, but also acknowledging the medical expertise and the doctor’s influence on one’s health can be auspicious.

Therefore, to fully understand the relationship between HLOC beliefs and adherence, examining solely the main effects of the sub-dimensions may not be sufficient, but looking into the interaction effects between Internal HLOC and External HLOC as well as External HLOC subdimensions on medical regimen adherence might be more fruitful [167,211]. Some studies recognized the importance of the interactional component of patient empowerment, therefore they addressed how the attitudinal symmetry in the doctor-patient dyads regarding patients’ control over health outcomes influenced medication adherence [212,213]. Indeed, patients who held highly similar beliefs to their physicians regarding a patients’ degree of control over their own health, adhered more to their medication regimen than patients who believed more strongly in their own personal control over their health than their doctor did [213].

More than half of the studies report no link between HLOC and medication adherence. Wallston suggested that HLOC does not operate alone to determine a behavior [44], and that other factors such as self-efficacy should be incorporated as a moderator [45]. This idea is compatible with the empowerment concept, which emphasizes that these constructs can jointly explain health behavior such as adherence. The findings seem robust and applicable across different cultures and countries. However, the North American sample may be considered overly representative in this review (close to 70%); therefore, an international or cultural comparison may not be well-grounded or representative here. Moreover, the majority of the sample being North American may have impacted findings, since the health care system in the United States is characterized by an advanced state of technology, private markets and pluralism [214]. Patients in the US are expected to decide about purchasing a health insurance plan choosing from various for-profit commercial insurance companies or from non-profit insurers [214] based on several factors (e.g. premium, deductible and out-of-pocket costs), which might foster patient empowerment compared to countries with a single nationwide system of health insurance.

### ‘Joint empowerment’ as a facilitator of adherence: Theoretical, operational and policy implications

The review suggests that a shift might be necessary in the conceptual as well as the operational ways of considering patient empowerment. The current literature offers two seemingly opposite approaches: conceiving empowerment as a relational [7] versus an intrapersonal concept [6]. Our findings suggest that in the medication adherence context the individual and relational aspects of empowerment should be viewed as the two sides of the same coin. We argue that patients need a high level of self-efficacy and internal health control beliefs, but they also have to be capable of sharing the control over their disease management with the physician. If we conceptualize empowerment in the above described manner, that would require further research to capture the relational empowerment as a dynamic feature of the doctor-patient relationship unfolding during the medical encounter. On the individual level, that would also require a shift from understanding empowerment as patient autonomy to a framework which would consider it as the patients’ perceived capability to cooperate effectively with the physician and sharing control with him/her. More research would be necessary to discover the right amount of control assigned to the patient and to the physician in the different contexts, i.e. in different conditions or stages of the illness, etc.

The theoretical shift suggested above would call for an operational revolution of empowerment, i.e. developing new measures about the patients’ beliefs about being able to collaborate and discuss issues with the physician, and asking for advice when necessary. As this definition of empowerment unavoidably presupposes the need of the patients’ ability to judge when they can act autonomously and when they would be better off asking for guidance from the physician, it raises the question of whether such a high level of empowerment is beneficial without the necessary health literacy skills [215].

To sum up, patient empowerment can promote medication adherence, but it requires a co-constructed sense of control in the doctor-patient dyad. This has implications for the clinical practice and policy making, i.e.instead of forcing or reinforcing the vision of an overly empowered patient (which can be burdensome for some patients [216,217] and demanding for many health care providers), an equilibrium should be established, in which both parties are interchangeably in control. This requires the health care providers facilitating patient empowerment (internal HLOC and self-efficacy), but also pointing out the importance of relying on the medical expertise of the physicians (Doctor HLOC) while choosing together the best possible treatment. As finding this equilibrium might be challenging, it requires building a strong collaborative doctor-patient relationship and continuous negotiation during the medical encounter. Moreover, patients’ level of health literacy should be taken into account and facilitated, as a recent paper showed that empowerment has a stronger link to better health status among the adequately health literate patients [218]. Most importantly, this approach has the capacity to reconcile the notion of an empowered patient and the shared decision making framework [219], without raising the dilemma of power or imbalanced control in the doctor-patient relationship.

## Limitations

This systematic review is not without limitations. First, our approach might not have captured other conceptualizations of empowerment existing in relevant literature. Second, no quantitative value can be presented to demonstrate the relationship between empowerment and medication adherence, as neither of the concepts is currently operationalized consistently across studies, which would allow us to conduct a meta-analysis. Thirdly, the medication adherence measures only rarely distinguish between the intentional and unintentional dimensions of non-adherence, possibly leading to a cancelling out of the relationship between empowerment and intentional non-adherence, with the inclusion of unintentional non-adherence. Studies using objective measures more often reported null findings compared to studies applying subjective adherence measures, introducing potential measurement bias to the analysis. Fourthly, the vast majority of the included studies had a cross-sectional correlational design, therefore we cannot draw any causal inference regarding the relationship between empowerment and adherence from them. Moreover, we included observational and interventional studies together in the systematic review, which introduces substantial heterogeneity. However, given the importance of findings emerging from interventional studies for the practical implications and insights gained in causal relationships, including intervention studies might merit particular prominence. Furthermore, we may unavoidably have missed unpublished studies or those that were not captured by the search strategy, potentially leading to publication bias. However, we put a great effort into avoiding publication bias with such preventative measures as searching without limiting by outcome [220]. The study samples were heterogeneous regarding characteristics such as medical condition, age and education, which may have affected the results. The applied quality assessment checklist did not recommend a cut-off score for low quality, thus all studies with different quality scores were included in the final analysis. Lastly, we included only papers published in the English language, and this might have resulted in missing some relevant work written in other languages.

## Conclusions

Our findings indicate that a balanced view would be the most beneficial for facilitating adherence, one alongside which the patients’ beliefs in their own capacity and control over their health is simultaneously present with their acknowledgment of the physician’s role in their disease management. The integration of the relational and individual nature of the construct to a so-called ‘joint empowerment’ approach enables us to address the question of control as a versatile component in a doctor-patient dyad.

## Supporting information

S1 Table PRISMA checklist. (DOCX)

S2 Table Sample characteristics. (DOC)

S3 Table Overview of the included studies. (DOCX)

S4 Table Quality assessment. (XLSX)
