# Supplementary material for: Is patient empowerment the key to promote adherence? A systematic review of the relationship between self-efficacy, health locus of control and medication adherence
Source: PLoS One. 2017 Oct 17;12(10):e0186458. doi: 10.1371/journal.pone.0186458 (PMC5645121; doi:10.1371/journal.pone.0186458)
Supplement: S1 Table — (DOCX) [file pone.0186458.s001.docx]

| **ID** | **Authors (year)** | **Locus of control 1: RIELCS 2: MHLOC 3: Other LOC** | | | **Self-efficacy 1: Adherence/Medication management 2: Disease management 3: General 4: Other** | | | | **Other empowerment measure** | **Adherence 1: Objective**  **2: Subjective 3: Mixed** | | | **Country** | **Disease** | **Relationship** | **Quality** |
| --- | --- | --- | --- | --- | --- | --- | --- | --- | --- | --- | --- | --- | --- | --- | --- | --- |
|  |  | **1** | **2** | **3** | **1** | **2** | **3** | **4** |  | **1** | **2** | **3** |  |  |  |  |
| 1 | Abbott, Dodd, & Webb (1996) |  | X |  |  |  |  |  |  |  | X |  | UK | Cystic fibrosis | External MHLOC is beneficial for adherence. | 12 |
| 2 | Ahmedani, Peterson, Wells, Rand & Williams (2013) |  | X |  |  |  |  |  |  | X |  |  | USA | Asthma | God attributed HLOC negatively, Physician attributed HLOC positively relates to adherence. | 13 |
| 3 | Altice, Mostashari & Friedland (2001) |  |  | X |  |  |  |  |  |  |  | X | USA | AIDS | There was no relationship between LOC and adherence. | 11 |
| 4 | Apter, Reisine, Affleck, Barrows & ZuWallack (1998) |  | X |  |  |  |  |  |  | X |  |  | USA | Asthma | There was no relationship between MHLOC and adherence. | 10 |
| 5 | Archiopoli, Ginossar, Wilcox, Avila, Hill & Oetzel (2016) |  |  |  | X |  |  |  |  |  | X |  | USA | HIV | Medication self-efficacy predicts medication adherence. | 13 |
| 6 | Arnsten, Li, Mizuno, Knowlton, Gourevitch, Handley, Knight & Metsch (2007) |  | X |  | X |  |  | X |  |  | X |  | USA | HIV | There was no relationship between MHLOC and adherence, but there was a positive relationship between adherence and self-efficacy. | 13 |
| 7 | Atkins & Fallowfield (2006) |  | X |  |  |  |  |  |  |  | X |  | UK | Breast cancer | Internal HLOC was beneficial for adherence. | 13 |
| 8 | Atkinson, Nilsson-Schönnesson, Williams & Timpson (2008) |  |  |  | X |  |  |  |  |  | X |  | USA | HIV | There was a positive relationship between self-efficacy and adherence. | 13 |
| 9 | Aversa & Kimberlin (1996) |  | X |  |  |  |  |  |  |  | X |  | USA | HIV | There is no relationship between MHLOC and adherence. | 11 |
| 10 | Bader, Kremer, Erlich-Trungenberger, Rojas, Lohmann, Deobald, Lochmann, Altmeyer & Brockmeyer (2006) |  |  | X |  |  |  |  |  |  | X |  | Germany | HIV | Internal HLOC promotes critical (non)-adherence. | 9 |
| 11 | Bane, Hughes, & McElnay (2006) |  | X |  |  |  |  |  | X |  |  | X | Ireland, UK | Cardiovascular disease | Interaction between the HLOC dimensions and competence is a strong predictor of adherence. | 11 |
| 12 | Barclay, Mason, Hinkin, Castellon, Reinhard, Marion & Levine (2007) |  | X |  | X |  |  |  |  | X |  |  | USA | HIV | Younger cohort: Lower level of adherence was linked to lower Internal HLOC, higher Chance HLOC and low treatment adherence self-efficacy.  Older cohort: Lower level of adherence was linked to lower internal locus of control and social support. | 12 |
| 13 | Bazargan, Barbre, & Hamm (1993) |  | X |  |  |  |  |  |  |  |  | X | USA | Black elderly | Low Internal HLOC was linked to non-adherence. | 13 |
| 14 | Begley, McLaws, Ross & Gold (2008) |  |  |  | X |  |  |  |  |  | X |  | Australia | HIV | There is a positive relationship between self-efficacy and adherence. | 13 |
| 15 | Bennett, Rowe & Katz (1998) |  |  |  |  |  |  | X |  |  | X |  | UK | Asthma | There is no relationship between self-efficacy and adherence. | 10 |
| 16 | Berglund, Lytsy, & Westerling (2013) |  | X |  |  |  |  |  |  |  | X |  | Sweden | Cardiovascular diseases | HLOC was a mediator between perceived treatment necessity and adherence. | 13 |
| 17 | Bogart, Gray-Bernhardt, Catz, Hartmann & Otto-Salaj (2002) |  |  |  | X |  |  |  |  |  | X |  | USA | HIV/AIDS | Self-efficacy mediates the relationship between social upward negative comparison and adherence. | 11 |
| 18 | Bolman, Arwert & Vollink (2011) |  |  | X | X |  |  |  |  |  | X |  | The Netherlands | Asthma | HLOC and self-efficacy were positively associated with adherence. Self-efficacy also had a mediator role. | 11 |
| 19 | Bosma, Vermeulen, Verschuuren, Erasmus & van der Bij (2011) |  |  |  | X |  |  |  |  | X |  |  | The Nether-lands | Lung transplant recipients | There was no relationship between self-efficacy and adherence. | 11 |
| 20 | Breaux-Shropshire, Brown, Pryor & Maples (2012) |  |  |  | X |  |  |  |  |  | X |  | USA | Hypertension | There is a positive relationship between self-efficacy and adherence. | 11 |
| 21 | Brown, Littlewood & Vanable (2013) |  |  |  | X |  |  |  |  |  | X |  | USA | HIV | Low adherence was linked with low self-efficacy. | 13 |
| 22 | Brus, van de Laar, Taal, Rasker & Wiegman (1999) |  |  |  | X |  |  |  |  | X |  |  | The Netherlands | Rheumatoid arthritis | There is a positive relationship between self-efficacy and adherence. | 13 |
| 23 | Budd, Hughes & Smith (1996) |  | X |  |  |  |  |  |  |  | X |  | UK | Schizophrenia | MHLOC and adherence are not related. | 12 |
| 24 | Burra, Chen, McIntyre, Grace, Blackmore & Stewart (2007) |  |  |  | X |  |  |  |  |  | X |  | Canada | Depression | Self-efficacy and adherence are positively associated. | 13 |
| 25 | Carpenter, DeVellis, Fisher, DeVellis, Hogan & Jordan (2010) |  |  |  | X |  |  |  |  |  | X |  | USA | Vasculitis | Self-efficacy and adherence are positively related. Self-efficacy also had a mediator role. | 13 |
| 26 | Catz, Kelly, Bogart, Benotsch & McAuliffe (2000) |  |  |  | X |  |  |  |  |  | X |  | USA | HIV | There was a positive relationship between self-efficacy and adherence. | 13 |
| 27 | Cha, Erlen, Kim, Sereika & Caruthers (2008) |  |  |  | X |  |  |  |  |  | X |  | USA | HIV | There was a positive relationship between self-efficacy and adherence. Self-efficacy also had a mediator role. | 13 |
| 28 | Chang, Compton, Almeter & Fox (2015) |  |  |  | X |  |  |  |  |  | X |  | USA | Older adults with chronic pain | Following a motivational interviewing, patients in the intervention condition  showed a significant increase in self-efficacy and reduction in the risk of prescription  opioid misuse. | 13 |
| 29 | Chao, Nau, Aikens & Taylor (2005) |  |  |  | X |  |  |  |  |  | X |  | USA | Diabetes | There is a positive relationship between self-efficacy and adherence. | 13 |
| 30 | Chen, Sheu, Chang, Wang & Huang (2010) |  |  |  |  | X |  |  |  |  | X |  | China | Asthma | There is a positive relationship between self-efficacy and adherence. | 12 |
| 31 | Chen, Tsai, Lin, Shih & Chen (2010) |  |  |  |  | X |  |  |  |  | X |  | Taiwan | Epilepsy | There was a positive relationship between self-efficacy and adherence. | 13 |
| 32 | Chesney, Ickovics, Chambers, Gifford, Neidig, Zwickl & Wu (2000) |  |  |  | X |  |  |  |  |  | X |  | USA | AIDS | There was a positive association between self-efficacy and adherence. | 13 |
| 33 | Cholowski & Cantwell (2007) |  |  |  |  |  |  |  | X |  | X |  | Australia | Heart failure | There is a positive relationship between self-regulation and adherence. | 10 |
| 34 | Christensen, Howren, Hillis, Kaboli, Carter, Cvengros & ... Rosenthal (2010) |  | X |  |  |  |  |  |  | X |  |  | USA | Co-morbiddiabetes mellitus and hypertension | The attitudinal symmetry between the physician and the patient regarding control is the most important predictor of adherence. | 13 |
| 35 | Christensen, Wiebe & Lawton (1997) |  | X |  |  |  |  |  |  | X |  |  | USA | Hemodialysis | Powerful others HLOC had a mediator role in explaining adherence. | 11 |
| 36 | Christensen, Wiebe, Benotsch & Lawton (1996) |  | X |  |  |  |  |  | X | X |  |  | USA | Hemodialysis | Competence was only beneficial for adherence if the person had low internal HLOC. | 13 |
| 37 | Clark & Dodge (1999) |  |  |  |  | X |  |  |  |  | X |  | USA | Ambulatory patients | Baseline self-efficacy predicted medication adherence at 4 months and 12 months follow-up. | 13 |
| 38 | Colbert, Sereika, & Erlen (2012) |  |  |  | X |  |  |  |  |  |  | X | USA | HIV/AIDS | There is a positive relationship between self-efficacy and adherence. | 13 |
| 39 | Cook, Emiliozzi., El-Hajj & McCabe (2010) |  |  |  | X |  |  |  |  |  | X |  | USA | Ulcerative colitis | There is a positive relationship between self-efficacy and adherence. | 6 |
| 40 | Cook, McCabe, Emiliozzi & Pointer (2009) |  |  |  | X |  |  |  |  |  | X |  | USA | HIV | There is a positive relationship between self-efficacy and adherence. | 13 |
| 41 | Corless, Wantland, Kirksey et al. (2012) |  |  |  |  |  | X |  |  |  | X |  | USA | HIV | There is a positive relationship between self-efficacy and adherence. | 9 |
| 42 | Cotton & Antill (1984) | X |  |  |  |  |  |  |  |  | X |  | Australia | Various diagnosis | Internal and external LOC did not distinguish between adherent and non-adherent patient groups. | 13 |
| 43 | Craig & Wright (2012) |  | X |  |  |  |  |  |  |  | X |  | Australia | People using prophylactic medication | Chance health locus of control is associated with non-adherence. | 8 |
| 44 | Criswell, Weber, Xu & Carter (2010) |  |  |  | X |  |  |  |  |  |  | X | USA | Hypertension | There is a positive relationship between self-efficacy and adherence. | 13 |
| 45 | Curtin, Walters, Schatell, Pennell, Wise & Klicko (2008) |  |  |  |  |  |  | X |  |  | X |  | USA | Chronic kidney disease | There was a positive relationship between self-efficacy and adherence. | 13 |
| 46 | Cvengros, Christensen, Hillis & Rosenthal (2007) |  | X |  |  |  |  |  |  |  | X |  | USA | Various diagnosis | Attitudinal symmetry between the physician and her patient regarding control is the most important predictor of adherence. | 13 |
| 47 | Darling, OlmsteadLund & Faircloug (2008) |  | X |  |  |  |  |  |  |  |  | X | USA | Bipolar disorder | Internal HLOC is positively linked to adherence. | 12 |
| 48 | Davis, Jandrisevits et al. (2012) | X |  |  |  |  |  |  |  |  | X |  | USA | Emergency department patients | Internal HLOC is positively associated with adherence. | 13 |
| 49 | de Guzman, Guevara, Guiang, Gutierrez, Habaluyas, Hizon & Idanan (2013) |  |  |  |  |  | X |  |  |  | X |  | Philippines | Mixed (Filipino elderly holding prescriptions) | There was no relationship between self-efficacy and adherence. | 9 |
| 50 | Denhaerynck, Abraham, Gourley, Drent, De Vleeschouwer, Papajcik & De Geest (2003) |  |  |  | X |  |  |  |  |  |  | X | Switzerland | Kidney trans-plantation | There is a positive relationship between self-efficacy and adherence. | 12 |
| 51 | Dewing, Mathews, Lurie, Kagee, Padayachee & Lombard (2015) |  |  |  | X |  |  |  |  | X |  |  | South Africa | HIV | Self-efficacy deficit is linked to non-adherence. | 13 |
| 52 | DiIorio, McCarty, DePadilla, Resnicow, Holstad, Yeager, Sharma, Morisky & Lundberg (2009) |  |  |  | X |  |  |  |  |  | X |  | USA | HIV/AIDS | There is a positive relationship between self-efficacy and adherence. Self-efficacy also had a mediator role. | 13 |
| 53 | Edworthy & Devins (1998) |  |  |  | X |  |  |  |  |  | X |  | Canada | Osteoarthritis | Self-efficacy had a mediator role in explaining adherence. | 12 |
| 54 | Elder, Ramamonjiarivelo, Wütshire, Piper, Horn, Gilbert, Hullett & Allison (2012) |  |  |  | X |  |  |  |  |  | X |  | USA | Hypertension | There is a positive relationship between self-efficacy and adherence. | 5 |
| 55 | Fransen, Mesters, Janssen, Knottnerus & Muris (2009) |  |  | X | X |  |  |  |  |  | X |  | The Nether-lands | Dyspepsia | Patients with high external locus of control and low self-efficacy were less likely to be adherent than patients with low external LOC and high self-efficacy. | 13 |
| 56 | Fraser, Hadjimichael,& Vollmer (2001) |  |  |  |  | X |  |  |  | X |  |  | USA | Multiple sclerosis (relapsing-remitting) | There is a positive relationship between self-efficacy and adherence. | 13 |
| 57 | Fraser, Hadjimichel & Vollmer (2003) |  |  |  |  | X |  |  |  | X |  |  | USA | Multiple sclerosis (progressive forms) | There is a positive relationship between self-efficacy and adherence. | 11 |
| 58 | Fraser, Morgante, Hadjimichel & Vollmer (2004) |  |  |  |  | X |  |  |  | X |  |  | USA | Multiple sclerosis | There is a positive relationship between self-efficacy and adherence.  Self-efficacy predicts adherence at 6-months follow-up. | 13 |
| 59 | Frazier, Davis-Ali & Dahl (1994) |  | X |  |  |  |  |  |  |  | X |  | USA | Renal transplant | External HLOC is negatively associated with adherence. | 12 |
| 60 | Fuertes, Boylan & Fontanella (2008) |  |  |  | X |  |  |  |  |  | X |  | USA | Patients with one or more chronic medical conditions | There is a positive relationship between self-efficacy and adherence. | 13 |
| 61 | Gastal, Pinheiro & Vazquez (2007) |  |  |  |  | X |  |  |  |  | X |  | Brazil | Diabetes | There is a positive relationship between self-efficacy and adherence. | 13 |
| 62 | Gatti, Jacobson, Gazmararian, Schmotzer & Kripalani (2009) |  |  |  | X |  |  |  |  |  | X |  | USA | General | There is a positive relationship between self-efficacy and adherence. | 13 |
| 63 | Gifford, Bormann, Shively, Wright, Richman & Bozzette (2000) |  |  |  | X |  |  |  |  |  | X |  | USA | HIV | There is a positive relationship between self-efficacy and adherence and self-efficacy had a mediator role. | 8 |
| 64 | Godin, Cotè, Naccache, Lambert & Trottier (2005) |  |  |  | X |  |  |  |  |  | X |  | Canada | HIV | There is a positive relationship between self-efficacy and adherence. | 13 |
| 65 | Graveley & Oseasohn (1991) |  | X |  |  |  |  |  |  | X |  |  | USA | Not specified. | There is no relationship between MHLOC and adherence. | 12 |
| 66 | Gremigni, Bacchi, Turrini, Cappelli,Albertazzi & Bitti (2007) |  |  |  |  |  |  |  | X |  | X |  | Italy | Renal transplant | There is a positive relationship between perceived autonomy and adherence. | 12 |
| 67 | Halimi, Vachier, Varrin, M., Godard, Pithon & Chanez (2007) | X |  |  |  |  |  |  | X |  | X |  | France | Asthma | Internal HLOC is positively linked to adherence. | 11 |
| 68 | Halimi, Pry, Pithon, Godard, Varrin & Chanez (2010) | X | X |  |  |  |  |  |  | X |  |  | France | Asthma | LOC is related to adherence, but MHLOC is not associated with adherence.  ILOC was more beneficial for adherence than ELOC. | 13 |
| 69 | Halkitis, Kutnick & Slater (2005) |  |  |  | X |  |  |  |  |  |  | X | USA | HIV | There is a positive relationship between self-efficacy and adherence. | 13 |
| 70 | Hargrave & Remler (1996) |  | X |  |  |  |  |  |  | X |  |  | USA | Epilepsy | There is no relationship between self-efficacy and adherence. | 10 |
| 71 | Heckman & Ellis (2011) |  |  |  |  | X |  |  |  |  | X |  | USA | Headache | Patients who had less headache management self-efficacy  were less likely to adhere  6 months later. | 12 |
| 72 | Hernandez-Tejada, Campbell, Walker, Smalls, Davis & Egede (2012) |  |  |  |  |  |  |  | X |  | X |  | USA | Diabetes | There is a positive relationship between diabetes empowerment and adherence. | 12 |
| 73 | Holloway, Rogers & Gershenhorn (1992) | X |  |  |  |  |  |  |  |  | X |  | USA | Not specified | External and Internal LOC did not explain adherence. | 12 |
| 74 | Hong, Oddone, Dudley & Bosworth (2006) |  | X |  |  |  |  |  |  |  | X |  | USA | Hypertension | Higher internal locus of control was associated with better medication adherence. Additionally, internal locus of control served as a moderator. | 12 |
| 75 | Johnson, Catz, Remien, Rotheram-Borus, Morin, Charlebois, Gore-Felton, Goldsten, Wolfe, Lightfoot, Chesney & NIMH Healthy Living Project Team (2003) |  |  |  | X |  |  |  |  |  | X |  | USA | HIV | There is a positive relationship between self-efficacy and adherence. | 13 |
| 76 | Johnson, Chesney, Goldstein, Remien, Catz, Gore-Felton, Charlebois, Morin & NIMH Healthy Living Project Team (2006) |  |  |  | X |  |  |  |  |  | X |  | USA | HIV | Self-efficacy had a mediator role in predicting adherence. | 13 |
| 77 | Johnson, Neilands,  Dilworth, Morin,  Remien & Chesney (2007) |  |  |  | X |  |  |  |  |  | X |  | USA | HIV | There is a positive relationship between self-efficacy and adherence. | 13 |
| 78 | Kalichman, Kalichman, Cherry, Swetzes, Amaral, White, Jones, Grebler & Eaton (2011) |  |  |  | X |  |  |  |  |  |  | X | USA | HIV/AIDS | There is a positive relationship between self-efficacy and adherence. | 13 |
| 79 | Kalichman, Rompa, DiFonzo, Simpson, Austin, Luke & Buckles (2001) |  |  |  | X |  |  |  |  |  | X |  | USA | HIV | There is a positive relationship between self-efficacy and adherence. | 13 |
| 80 | Kamau, Olson, Zipp & Clark (2011) |  |  |  |  |  |  | X |  |  | X |  | Kenya | HIV | There is a positive relationship between self-efficacy and adherence. | 13 |
| 81 | Kamolz (2002) |  | X |  |  |  |  |  |  |  | X |  | Austria | Gastro-esophageal reflux disease | Chance HLOC is negatively associated with adherence. | 9 |
| 82 | Katerndahl (2001) |  | X |  |  |  |  |  |  |  |  | X | USA | Various diagnosis | No relationship was found between MHLOC and adherence. | 10 |
| 83 | Kaya, Erkan, Ozkan, Ozkan,Kocaman, Ertekin & Direk (2009) | X |  |  |  |  |  |  |  |  | X |  | Turkey | Asthma | There was no relationship between the RIELCS score and compliance at the 3 month follow-up. At the 6 month follow-up, the patients with higher external locus of control had better compliance than patients with higher Internal LOC. | 10 |
| 84 | Kennedy, Goggin & Nollen (2004) |  |  |  |  |  |  |  | X |  | X |  | USA | HIV | Perceived competence is identified as the strongest direct predictor of adherence, and is associated with patients’ sense of autonomy regarding their medication adherence. | 13 |
| 85 | Kerr, Marshall, Walsh, Palepu, Tyndall, Montaner & Wood (2005) |  |  |  | X |  |  |  |  |  |  | X | Canada | HIV | There is a positive relationship between self-efficacy and adherence. | 13 |
| 86 | Khdour , Hawwa, Kidney, Smyth & McElnay (2012) |  |  |  |  | X |  |  |  |  | X |  | Ireland | Chronic obstructive pulmonary disease | There is a positive relationship between self-efficacy and adherence. | 11 |
| 87 | Kim, Kim, Kim, Park, Chung & Chu (2011) |  |  |  |  | X |  |  |  |  | X |  | Korea | Anti-coagulation/ thrombus/ atrial fibrillation | There is a positive relationship between self-efficacy and adherence. | 13 |
| 88 | Lamba, Nagurka, Desai, Chun, Holland & Koneru (2012) |  |  | X |  |  |  |  |  |  | X |  | USA | Liver transplant | No relationship between Healthcare LOC and adherence. | 13 |
| 89 | Lewis, Schoenthaler & Ogedegbe (2012) |  |  |  | X |  |  |  |  |  | X |  | USA | Hypertension | There is a positive relationship between self-efficacy and adherence. | 10 |
| 90 | Li, Huang, Wang, Fennie, He & Williams (2011) |  |  |  |  | X |  |  |  |  | X |  | China | HIV | There is a positive relationship between self-efficacy and adherence. | 13 |
| 91 | Liu, Malin, Diamant,Thind & Maly (2012) |  |  |  |  |  |  | X |  |  | X |  | USA | Breast cancer | There is a positive relationship between self-efficacy and adherence. | 12 |
| 92 | Luszczynska, Sarkar & Knoll (2007) |  |  |  |  |  | X |  |  |  |  | X | India | HIV | There is a positive relationship between self-efficacy and adherence and self-efficacy also served as a mediator. | 13 |
| 93 | Lynam, Catley, Goggin., Rabinowitz, Gerkovich, Williams, Wright & MOTIV8 (2009) |  | X |  | X |  |  |  | X | X |  |  | USA | HIV | There is a positive relationship between self-efficacy and adherence. HLOC is suggested to be important as a precursor of a one’s self-efficacy. | 13 |
| 94 | Mann, Ponieman, Leventhal & Halm (2009) |  |  |  |  | X |  |  |  |  | X |  | USA | Diabetes | There is a positive relationship between self-efficacy and adherence. | 13 |
| 95 | MacDonell, Jacques-Tiura, Naar, Fernandez & ATN 086/106  Protocol Team |  |  |  | X |  |  |  |  |  | X |  | USA | HIV/AIDS | High self-efficacy predicted high adherence. | 13 |
| 96 | Marc , Testa, Walker, Robbins, Shafer, Anderson & Berkman (2007) |  |  |  | X |  |  |  |  |  | X |  | USA Italy | HIV | There is a positive relationship between self-efficacy and adherence and self-efficacy also served as a mediator. | 12 |
| 97 | McDonald-Miszczak, Maki, & Gould (2000) |  | X |  |  |  |  |  |  |  | X |  | USA | Not specified | People who have a high Powerful others HLOC are less adherent compared to others with lower Powerful Others HLOC. | 13 |
| 98 | McDonough, Boyd, Varvares & Maves (1996) |  | X |  |  |  |  |  |  |  | X |  | USA | Head and neck cancer | There is no relationship between MHLOC and adherence. | 11 |
| 99 | Mishali, Omer & Heymann (2011) |  |  |  | X |  |  |  |  |  | X |  | Israel | Diabetes | There is a positive relationship between self-efficacy and adherence. | 13 |
| 100 | Mo & Mak (2009) |  |  |  | X |  |  |  |  |  | X |  | Hong Kong | HIV/AIDS | There is a positive relationship between self-efficacy and adherence. | 13 |
| 101 | Mohr, Boudewyn, Likosky, Levine & Goodkin (2001) |  |  |  |  | X |  |  |  |  |  | X | USA | Multiple sclerosis | There is a positive relationship between self-efficacy and adherence. Self-efficacy served also as a mediator. | 13 |
| 102 | Molassiotis, Nahas-Lopez, Chung, Lam, Li & Lau (2002) |  | X |  | X |  |  |  |  |  | X |  | Hong Kong | HIV | High self-efficacy and internal locus of control had a positive association with adherence. | 13 |
| 103 | Molassiotis, Morris & Trueman (2007) |  |  |  |  |  |  |  | X |  | X |  | UK | HIV | There is a weak positive relationship between treatment-related empowerment and adherence. | 12 |
| 104 | Molloy, Randall, Wikman, Perkins-Porras, Messerli-Bürgy, & Steptoe (2012) |  |  |  |  | X |  |  |  |  | X |  | UK | Coronary heart disease | There is a positive relationship between self-efficacy and adherence. Self-efficacy served as a mediator. | 13 |
| 105 | Morasco et al. (2013) |  |  |  |  | X |  |  |  |  | X |  | USA | Chronic pain | There is a positive relationship between self-efficacy and adherence. | 13 |
| 106 | Murphy, Greenwell & Hoffman (2002) |  |  |  | X |  |  |  |  |  |  | X | USA | HIV/AIDS | There is a positive relationship between self-efficacy and adherence. | 10 |
| 107 | Myers & Myers (1999) |  | X |  |  |  |  |  |  |  | X |  | UK | Cystic fibrosis | Powerful others, especially Doctor HLOC is positively linked to medication adherence. | 9 |
| 108 | Naar-King, Templin, Wright, Frey, Parsons & Lam (2006) |  |  |  | X |  |  |  |  |  | X |  | USA | HIV | There is a positive relationship between self-efficacy and adherence. | 12 |
| 109 | Náfrádi, Galimberti, Nakamoto & Schulz (2016) |  |  |  | X |  |  |  |  |  | X |  | Italian-speaking area of Switzerland/Italy | Hypertension | Adherence self-efficacy is associated with medication adherence. | 13 |
| 110 | Nokes, Johnson et al. (2012) |  |  |  | X |  |  |  |  |  | X |  | USA Puerto Rico | HIV | There is a positive relationship between self-efficacy and adherence. | 13 |
| 111 | O’Hea, Moon, Grothe, Boudreaux, Bodenlos, Wallston & Brantley (2009) |  | X |  |  | X |  |  |  | X |  |  | USA | Diabetes | A three way interaction among the perceived control measures was associated with adherence. | 13 |
| 112 | O'Hea, Grothe, Bodenlos, Boudreaux, White & Brantley (2005) |  | X |  |  |  |  |  |  | X |  |  | USA | Diabetes | Only Internal HLOC was DIRECTLY related to adherence. The interactions between HLOC dimensions were significantly related to adherence. | 13 |
| 113 | Parsons, Rosof & Mustanski (2008) |  |  |  | X |  |  |  |  |  | X |  | USA | HIV | There is a positive relationship between self-efficacy and adherence. | 13 |
| 114 | Pepper, Carpenter & DeVellis (2012) |  |  |  | X |  |  |  |  |  | X |  | USA | Vasculitis | There is a positive relationship between self-efficacy and adherence. Self-efficacy also had a mediator role. | 12 |
| 115 | Peyrot & Rubin (1994) |  |  | X |  |  |  |  |  |  | X |  | USA | Diabetes | Internal DLC was divided into self-blame and autonomy. Autonomy was significantly associated with positive outcomes, but self-blame was related to negative outcomes. Regarding Powerful others DLC, infrequent insulin dose adjustment was associated positively with Health Professional LOC, but infrequent late shots were correlated with Nonmedical Other DLC. Chance DLC was associated with dysfunctional disease management measures. | 12 |
| 116 | Pinheiro, de-Carvalho-Leite, Drachler & Silveira (2002) |  |  |  | X |  |  |  |  |  | X |  | Brazil | HIV | There is a positive relationship between self-efficacy and adherence. | 13 |
| 117 | Ponieman, Wisnivesky, Leventhal, Musumeci-Szabó & Halm (2009) |  |  |  |  |  | X | X |  |  | X |  | USA | Asthma | The behavior-specific self-efficacy is more important predictor of adherence than generic self-efficacy. | 13 |
| 118 | Raiz, Kilty, Henry & Ferguson (1999) |  | X |  |  |  |  |  |  |  | X |  | USA | Renal transplant patients | High Powerful others HLOC was associated with medication adherence, while high Internal HLOC was associated with lower medication adherence. | 13 |
| 119 | Reach, Michault, Bihan, Paulino, Cohen & Le Clésiau (2011) |  | X |  |  |  |  |  |  |  |  | X | France | Diabetes | Chance HLOC and Powerful others HLOC was negatively related to adherence. | 13 |
| 120 | Remien, Bastos, Tertor, Raxach, Pinto, Parker, Berkman & Hacker (2007) |  |  |  | X |  |  |  |  |  | X |  | Brazil | HIV | There is a positive relationship between self-efficacy and adherence. | 12 |
| 121 | Resnick, Wehren & Orwig (2003) |  |  |  | X |  |  |  |  |  | X |  | USA | Osteoporosis | There is a positive relationship between self-efficacy and adherence. | 13 |
| 122 | Robbins, D’Aquila, Morgello, Byrd, Remien, Mindt & Rivera (2012) |  |  |  |  |  |  | X |  | X |  |  | USA | HIV | There is a positive relationship between self-efficacy and adherence. | 12 |
| 123 | Russell, Cetingok, Hamburger, Owens, Thompson, Hathaway, Winsett, Conn, Madsen, Sitler & Wakefield (2010) |  |  |  | X |  |  |  |  |  |  | X | USA | Renal transplant patients | There was no relationship between self-efficacy and adherence | 13 |
| 124 | Safren, Otto, Worth, Salomon, Johnson, Mayer & Boswell (2001) |  |  |  | X |  |  |  |  |  | X |  | USA | HIV | There is a positive relationship between self-efficacy and adherence. | 11 |
| 125 | Sajatovic, Ignacio, West, Cassidy, Safavi, Kilbourne & Blow (2009) |  | X |  |  |  |  |  |  |  | X |  | USA | Bipolar disorder | Non-adherent individuals had higher Powerful others HLOC score compared to adherent individuals. | 10 |
| 126 | Sajatovic, Micula-Gondek, Tatsuoka & Bialko (2011) |  | X |  |  |  |  |  |  |  | X |  | USA | Bipolar disorder | Powerful others HLOC was associated with greater adherence. | 13 |
| 127 | Samal et al. (2011) |  |  |  |  | X |  |  |  |  | X |  | USA | HIV/AIDS | Self-efficacy does not mediate the relationship between information seeking and adherence. | 9 |
| 128 | Sarkar,Fisher & Schillinger (2006) |  |  |  |  | X |  |  |  |  | X |  | USA | Diabetes | There was no relationship between self-efficacy and adherence. | 11 |
| 129 | Scherer & Bruce (2001) |  |  |  |  | X |  |  |  |  | X |  | USA | Asthma | There is a positive relationship between self-efficacy and adherence. | 12 |
| 130 | Schneider (1992) |  | X |  |  |  |  |  |  | X |  |  | USA | Hemodialysis | Powerful Others HLOC is negatively associated with adherence. | 13 |
| 131 | Schneider, Wensing, Quinzler, Bieber & Szecsenyi (2007) |  |  |  |  |  |  |  | X |  | X |  | Germany | Asthma | Higher autonomy preference fosters motivation for self-management but is negatively linked to medication adherence. | 13 |
| 132 | Schoenthaler, Ogedegbe & Allegrante (2009) |  |  |  | X |  |  |  |  |  | X |  | USA | Cardio-vascular disease | There was a positive relationship between self-efficacy and adherence. Self-efficacy also was a mediator. | 13 |
| 133 | Schousboe, Dowd, Davison & Kane (2010) |  |  |  | X |  |  |  |  |  | X |  | USA | Osteoporosis | There was a positive relationship between self-efficacy and adherence. | 13 |
| 134 | Sevelius, Carrico & Johnson (2010) |  |  |  | X |  |  |  |  |  | X |  | USA | HIV | The integration subscale of self-efficacy was positively linked to medication adherence. | 13 |
| 135 | Shively, Smith, Bormann & Gifford (2002) |  |  |  | X |  |  | X |  |  | X |  | USA | HIV | There is a positive relationship between self-efficacy and adherence. | 10 |
| 136 | Shon & Park (2002) |  |  |  | X |  |  |  |  |  | X |  | Korea | Psychiatric disorder | There is a positive relationship between self-efficacy and adherence. | 11 |
| 137 | Simoni, Frick & Huang (2006) |  |  |  | X |  |  |  |  |  | X |  | USA | HIV/AIDS | Self-efficacy at baseline predicted adherence at 3 months. | 13 |
| 138 | Simoni, Frick, Lockhart & Liebovitz (2002) |  |  |  | X |  |  |  |  |  | X |  | USA | HIV | There was a positive relationship between self-efficacy and adherence. Self-efficacy also was a mediator. | 8 |
| 139 | Stanton (1987) |  | X | X |  |  |  |  |  |  |  | X | USA | Hypertension | Greater expectancy for internal control over health and hypertension were significant determinants of adherence. | 11 |
| 140 | Theofilou (2013) |  | X |  |  |  |  |  |  |  | X |  | Greece | Hemodialysis | Patients, who were more adherent to their medical regimen, had high scores on Internal and Doctor HLOC, and low scores on Powerful others HLOC. | 13 |
| 141 | Unni & Farris (2011) |  |  |  | X |  |  |  |  |  | X |  | USA | Cholesterol Asthma | There was a positive relationship between self-efficacy and adherence. | 13 |
| 142 | Valeberg, Miaskowski, Hanestad, Bjordal, Moum & Rustøen (2008) |  |  |  |  |  |  | X |  |  | X |  | Norway | Oncology outpatients with analgesic medication | Patients with lower self-efficacy scores for physical function reported higher adherence scores. | 13 |
| 143 | Voils, Steffens, Flint & Bosworth (2005) |  | X |  |  |  |  |  |  |  | X |  | USA | Depression | HLOC moderated the relationship between social support and adherence. | 13 |
| 144 | Wang, Bohn, Knight, Glynn, Mogun & Avorn (2002) | X |  |  |  |  |  |  |  | X |  |  | USA | Hypertension | There was a trend towards better compliance among patients with External LOC. | 13 |
| 145 | Warren-Findlow, Seymour & Huber (2012) |  |  |  |  | X |  |  |  |  | X |  | USA | Hypertension | There was a positive relationship between self-efficacy and adherence. | 10 |
| 146 | Warren-Findlow, Seymour & Shenk (2011) |  |  |  |  | X |  |  |  |  | X |  | USA | Hypertension | Having good self-efficacy to manage hypertension was associated with 2.5 times better odds of being adherent. | 13 |
| 147 | Weng, Israni, Joffe, Hoy, Gaughan, Newman & Feldman (2005) |  | X |  |  |  |  |  |  | X |  |  | USA | Renal transplan-tation | In the unadjusted regression model, Powerful Others HLOC predicted with adherence. In the adjusted model, Powerful others HLOC was not a significant predictor of adherence. | 13 |
| 148 | Williams, Patrick, Niemiec,, Williams, Divine, Lafata, Heisler , Tunceli & Pladevall (2009) |  |  |  |  |  |  |  | X |  |  | X | USA | Diabetes | Autonomous self-regulation for medication use had a positive link to perceived competence.  Perceived competence was positively associated with medication adherence. | 13 |
| 149 | Williams, Rodin, Ryan, Grolnick & Deci (1998) |  | X |  |  |  |  |  | X |  | X |  | USA | Outpatients with various diseases | Patients' autonomous motivation is a mediator for predicting adherence, but no relationship is found between HLOC and adherence. | 13 |
| 150 | Wilson, Doxanakis & Fairley (2004) |  |  |  | X |  |  |  |  |  | X |  | Australia | HIV | There was a positive relationship between self-efficacy and adherence. | 12 |
| 151 | Wolf, Davis, Osborn, Skripkauskas, Bennett & Makoul (2007) |  |  |  | X |  |  |  |  |  | X |  | USA | HIV | There was a positive relationship between self-efficacy and adherence. | 13 |
| 152 | Wulandari,Craig & Whelan (2013) |  |  | X |  |  |  |  |  |  | X |  | Indonesia | Pregnancy | Internal HLOC is beneficial for adherence. By contrast, Chance HLOC is negatively associated with adherence. | 10 |
| 153 | Yu, Yeoh, Seow, Luo & Griva (2012) |  |  |  |  |  |  |  |  |  |  | X | Singapore | End-stage renal disease | No relationship between self-efficacy and adherence. | 8 |
| 154 | Zwibel, Pardo, Smith, Denney & Oleen-Burkey (2010) |  |  |  |  | X |  |  |  |  |  | X | USA | Multiple sclerosis | Self-efficacy was a significant predictor of adherence in case of treatment naive patients, but not in case of treatment experienced patients. | 11 |

* **RIELCS**: Rotter’s Internal-External Locus of Control Scale; **MHLOC**: Multidimensional Health Locus of Control; **LOC**: Locus of Control
