# Supplementary material for: Is patient empowerment the key to promote adherence? A systematic review of the relationship between self-efficacy, health locus of control and medication adherence
Source: PLoS One. 2017 Oct 17;12(10):e0186458. doi: 10.1371/journal.pone.0186458 (PMC5645121; doi:10.1371/journal.pone.0186458)
Supplement: S3 Table — (DOCX) [file pone.0186458.s003.docx]

| **ID** | **Authors (year)** | **Sample size** | **Gender** | **Mean age** | **Race/Ethnicity** |
| --- | --- | --- | --- | --- | --- |
| 1 | Abbott, Dodd, & Webb (1996) | N=60 | 25 female, 35 male | 20.98 years  (range 16-44) | Ethnicity: N.A. |
| 2 | Ahmedani, Peterson, Wells, Rand & Williams (2013) | N=1025 | 675 female, 350 male | 37.59 years | White (n=702)  African American (n=323) |
| 3 | Altice, Mostashari & Friedland (2001) | N=205 | 98 female, 107 male | 36.2 years | Black (n=84)  White (n=19)  Hispanic (n=40) |
| 4 | Apter, Reisine, Affleck, Barrows & ZuWallack (1998) | N=50 | 37 female, 13 male | 46+/-14 yr, with a  range of 20 to 81 yr | African–American (n=11)  Hispanic (n=18)  Caucasian (n=21) |
| 5 | Archiopoli, Ginossar, Wilcox, Avila, Hill & Oetzel (2016) | N = 344 | 35 female, 303 male | 48 (median age) | White, non-Hispanic (179) Hispanic (111)  American Indian (13)  Multi-ethnic (18)  Other (African-American, Asian-Pacific  Islander, Other)  (20) |
| 6 | Arnsten, Li, Mizuno, Knowlton, Gourevitch, Handley, Knight & Metsch (2007) | N=1161 | 747 female, 414 male | > 40 years (399) | Non-Hispanic white 43 (7)  Non-Hispanic black 412 (66)  Hispanic 124 (20)  Non-Hispanic other 42 (7) |
| 7 | Atkins & Fallowfield (2006) | N=208 | women | 59.40 (SD 11.53), range 56 (32–88) | N.A. |
| 8 | Atkinson, Nilsson-Schönnesson, Williams & Timpson (2008) | N=130 | 74% male | Average age of 40.0 years (SD=6.8 years) | African-Americans |
| 9 | Aversa & Kimberlin (1996) | N=98 | 28 female, 74 male | 21-30 years (21)  31-40 years (51)  41-54 years (24) | White (69)  Black (24)  Hispanic (4) |
| 10 | Bader, Kremer, Erlich-Trungenberger, Rojas, Lohmann, Deobald, Lochmann, Altmeyer & Brockmeyer (2006) | N=100 | 35 female, 65 male | 20-30 years (6)  31-40 years (45)  41-50 years (33)  >50 years (16) | N.A. |
| 11 | Bane, Hughes, & McElnay (2006) | N=122 | 49 female, 73 male | 54.51 years (12.47) | N.A. |
| 12 | Barclay, Mason, Hinkin, Castellon, Reinhard, Marion & Levine (2007) | N=185 | 41 female, 144 men | 44.01 (sd=7.3) | African American (127)  Caucasian (32)  Hispanic (18)  Asian (2)  American Indian (1)  Multiracial (5) |
| 13 | Bazargan, Barbre, & Hamm (1993) | N=571 | N.A. | >62 years | Black elderly |
| 14 | Begley, McLaws, Ross & Gold (2008) | N=179 | 3 female, 176 male | 40.5  years (SD¼8.3, range¼22–62 years) | N.A. |
| 15 | Bennett, Rowe & Katz (1998) | N=71 | 40 female, 31 male | 47 years (SD = 19.25) | N.A. |
| 16 | Berglund, Lytsy, & Westerling (2013) | N=414 | 49.2% female, 50.8% male | 64.2 (SD=9.5) | N.A. |
| 17 | Bogart, Gray-Bernhardt, Catz, Hartmann & Otto-Salaj (2002) | N=44 | 7 female, 37 male | N.A. | White (19)  African American (50%)  Hispanic (2)  Native American (1) |
| 18 | Bolman, Arwert & Vollink (2011) | N=139 | 98 female, 41 male | 31.5 (SD=5.6) | N.A. |
| 19 | Bosma, Vermeulen, Verschuuren, Erasmus & van der Bij (2011) | N=105 | 63 female, 42 male | median age= 54 (range: 19–66) | 96.7% were Caucasians |
| 20 | Breaux-Shropshire, Brown, Pryor & Maples (2012) | N=149 | male (85%) | 47 years | Black (71%) |
| 21 | Brown, Littlewood & Vanable (2013) | N=116 | 42% female | 45.4 years | 43% African American |
| 22 | Brus, van de Laar, Taal, Rasker & Wiegman (1999) | N=65 | 44 female, 16 male | 59 years | N.A. |
| 23 | Budd, Hughes & Smith (1996) | N.A. | N.A. | N.A. | N.A. |
| 24 | Burra, Chen, McIntyre, Grace, Blackmore & Stewart (2007) | N=80 | 42 female, 38 male | 63 years | 77 Caucasian |
| 25 | Carpenter, DeVellis, Fisher, DeVellis, Hogan & Jordan (2010) | N=228 | female (70%) | 51 years | White (91%) |
| 26 | Catz, Kelly, Bogart, Benotsch & McAuliffe (2000) | N=72 | 9 female, 63 male | 40 years (range: 24-61) | 36% African American  56% Caucasian |
| 27 | Cha, Erlen, Kim, Sereika & Caruthers (2008) | N=215 | 70 female, 145 male | 40.7 years, SD=7.58  (range: 19-61) | White (n = 134) |
| 28 | Chang, Compton, Almeter & Fox (2015) | N=30 | 15 female | 59.1 (SD = 6.1) | Black (63.3%) |
| 29 | Chao, Nau, Aikens & Taylor (2005) | N=445 | Females and males (50.1% vs 49.9%) | 56.3 years (SD, 11.4 years) | 78.2% Caucasian |
| 30 | Chen, Sheu, Chang, Wang & Huang (2010) | N=60 | 38 female, 22 male | Experimental group: 52.2 years (s.d. =12.74)  Control group: 53.97 years (s.d.=13.64) | N.A. |
| 31 | Chen, Tsai, Lin, Shih & Chen (2010) | N=357 | 164 female, 193 male | 38.3 years (sd=11.4 years) | N.A. |
| 32 | Chesney, Ickovics, Chambers, Gifford, Neidig, Zwickl & Wu (2000) | N=75 | 20% female, 80% male | 39 years (range 20-72) | White (69%)  African-American (11%)  Hispanic (9%)  Other (11%) |
| 33 | Cholowski & Cantwell (2007) | N=54 | 20 female, 33 male, 1 person: N.A. | 72.33 (SD=8.19) | N.A. |
| 34 | Christensen, Howren, Hillis, Kaboli, Carter, Cvengros & ... Rosenthal (2010) | N=224 | Male: 224 (100%) | 68.8 (9.7) | N.A. |
| 35 | Christensen, Wiebe & Lawton (1997) | N=48 | 22 female, 26 male | 56.2 | N.A. |
| 36 | Christensen, Wiebe, Benotsch & Lawton (1996) | N=81 | 41 female, 40 male | 54.85 | N.A. |
| 37 | Clark & Dodge (1999) | N=485 | 485 female | 71.8 | 87% Caucasian  African American 11.6%  Hispanic 0.7%  Asian 0.4%  Other 0.3%. |
| 38 | Colbert, Sereika, & Erlen (2012) | N=302 | 89 female, 213 male | 20–30 years: 28  31–54 years: 261  55 and up:23 | 125 White  177 African American |
| 39 | Cook, Emiliozzi., El-Hajj & McCabe (2010) | N=524 | 359 female, 165 male | 49 years (SD = 17.7) | N.A. |
| 40 | Cook, McCabe, Emiliozzi & Pointer (2009) | N=98 | 38 women | 43.5 years (sd=9.3) | N.A. |
| 41 | Corless, Wantland, Kirksey et al. (2012) | N=569 | 218 female, 340 male, 11 transgender | 42.8 years (SD = 9.6) | 227 African/African American  102 Caucasian  195 Hispanic  10 Asian/ Pacifica Islander  30 Other |
| 42 | Cotton & Antill (1984) | N=109 | 81 female, 28 male | 52 years | N.A. |
| 43 | Craig & Wright (2012) | N=65 | 41 female, 24 male | 48.62 years (range: 15.1) | N.A. |
| 44 | Criswell, Weber, Xu & Carter (2010) | N=584 | 340 female, 244 male | Intervention group: 58.2 (sd:14.2)  Control group: 59.9 (sd: 13.2) | 493 Caucasian |
| 45 | Curtin, Walters, Schatell, Pennell, Wise & Klicko (2008) | N=174 | 42% male | 50.89 years (sd: 13.57) | 12.1% White  64.4% Black |
| 46 | Cvengros, Christensen, Hillis & Rosenthal (2007) | 16 primary care physicians from a single academic medical  center and 146 patients | The physician sample was 55% female, and the patient  sample was 65% female. | The average age of physicians and patients was 56.0 (SD ¼ 17.0) and 44.8 (SD ¼ 6.7),  respectively | N.A. |
| 47 | Darling, OlmsteadLund & Faircloug (2008) | N=100 | female (60%) | 42.8 years | White  (86%) |
| 48 | Davis, Jandrisevits et al. (2012) | N=472 | Male gender 50.1% | 50.2 years | Caucasian 46.5%  Black 30.8%  Hispanic 14.6%  Other 8.2% |
| 49 | de Guzman, Guevara, Guiang, Gutierrez, Habaluyas, Hizon & Idanan (2013) | N=325 | 201 female, 124 male | 61-70 years (n=202)  71-80 (n=85)  81-91 (n=38) | N.A. |
| 50 | Denhaerynck, Abraham, Gourley, Drent, De Vleeschouwer, Papajcik & De Geest (2003) | N=1021 | 78.2% male | 53.7 years (SD=14) | N.A. |
| 51 | Dewing, Mathews, Lurie, Kagee, Padayachee & Lombard (2015) | N=600 (300 adherent, 300 non-adherent) | 420 female, 180 male | adherent: 36.4 (8.2)  non-adherent: 35.3 (9.1) | N.A. |
| 52 | DiIorio, McCarty, DePadilla, Resnicow, Holstad, Yeager, Sharma, Morisky & Lundberg (2009) | N=236 | male (66.5%) | 40.98 years (SD = 7.125) | Black/African American (87.3%) |
| 53 | Edworthy & Devins (1998) | N=252 (Intervention n=126, Control n=126) | control: 76.2% female  intervention: 61.4% female | control: 63.8 years  intervention: 62.6 years | N.A. |
| 54 | Elder, Ramamonjiarivelo, Wütshire, Piper, Horn, Gilbert, Hullett & Allison (2012) | N=235 | males | <34 n=8  35-44 n=26  45-54 n=93  55-64 n=87  >65 n=21 | African American |
| 55 | Fransen, Mesters, Janssen, Knottnerus & Muris (2009) | N=347 | 58% male | 54years (SD 14) | N.A. |
| 56 | Fraser, Hadjimichael,& Vollmer (2001) | N=341  Group 1 (n=225)  Group 2 (n=116) | Group1: Female 84%  Group 2: Female 92.2% | Group 1: 42 (SD 8.57)  Group 2: 43 (SD 9.35) | Group1: White 89.3%, African American 4.9%, Other 5.7%  Group2: White 88.8%, African American 3.4%, Other 7.7% |
| 57 | Fraser, Hadjimichel & Vollmer (2003) | N=199 | group1: female (82%)  group2: female (70%) | 46 years | group 1: Caucasian: 86.9%, African American: 3.7%, Other: 5.6%  group 2: Caucasian: 85.9%, African American: 4.3%, Other: 7.6% |
| 58 | Fraser, Morgante, Hadjimichel & Vollmer (2004) | N=104 | group1: 43 years  group2: 45 years | group1: female (90%)  group2: female (86%) | group1: Caucasian (93%)  group2: Caucasian (91%) |
| 59 | Frazier, Davis-Ali & Dahl (1994) | N=241 | male: 58% | 42 years (sd=13.7 yrs) | Caucasian (91%) |
| 60 | Fuertes, Boylan & Fontanella (2008) | N=152 | 81 men,  71 women | 45.1 years (SD=12.75) | , 33 were Euro-American, 66 African-American, 44  Hispanic, 6 Asian-American, and 3 did not specify their race. |
| 61 | Gastal, Pinheiro & Vazquez (2007) | N=213 | 47.9% men  52.1% women | 33.9 ±  14.97 years | fair-skinned  (90.1%). |
| 62 | Gatti, Jacobson, Gazmararian, Schmotzer & Kripalani (2009) | N=275 | women  (73.1%) | 53.9 years | African  American (86.2%) |
| 63 | Gifford, Bormann, Shively, Wright, Richman & Bozzette (2000) | N=133 | 114 male, 19 female | 18-34 years (34)  35-49 (75)  above 50 (24) | Non-Hispanic white (83)  Non-Hispanic African American (29)  Hispanic (15)  Other (6) |
| 64 | Godin, Cotè, Naccache, Lambert & Trottier (2005) | N=376 | male (95.5%) | 42.9 (9/8.2) years | N.A. |
| 65 | Graveley & Oseasohn (1991) | N=249 | N.A. | N.A. | Anglo (134)  Non-Anglo (115)  Hispanic (89)  17 Black  9 Asian |
| 66 | Gremigni, Bacchi, Turrini, Cappelli,Albertazzi & Bitti (2007) | N= 34 | 38% male | 49 yrs (12) | N.A. |
| 67 | Halimi, Vachier, Varrin, M., Godard, Pithon & Chanez (2007) | N=73 | Group1: 11 male, 13 female  Group 2: 20 male, 29 female | Group1: 50yrs  Group2: 47yrs | N.A. |
| 68 | Halimi, Pry, Pithon, Godard, Varrin & Chanez (2010) | N=52 | Group1: 13 male, 16 female  Group 2: 10 male, 21 female | Group1: 56yrs  Group2: 47yrs | N.A. |
| 69 | Halkitis, Kutnick & Slater (2005) | N=300 | biological males | 42 (SD = 7.72) | 108 African American  62 Latino/Hispanic,  101 White  29 other or mixed race |
| 70 | Hargrave & Remler (1996) | N=31 | males | Group 1: 46.3 years [SD] 9.6])  Group 2: 56.4 years; SD 11.5) | Black |
| 71 | Heckman & Ellis (2011) | N=284 | female  (89%) | 36.6 years of age (SD = 10.4) | Pretreatment: Caucasians (n = 173),  African Americans (n = 112)  Follow-up: Caucasians (n = 79), African  Americans  (n = 32) |
| 72 | Hernandez-Tejada, Campbell, Walker, Smalls, Davis & Egede (2012) | N=378 | Female (69%), | between the ages of 50 and 64 years (54%) | Non-Hispanic blacks (83%), |
| 73 | Holloway, Rogers & Gershenhorn (1992) | N=148 | 51.2% women | 38 years | N.A. |
| 74 | Hong, Oddone, Dudley & Bosworth (2006) | N=588 | 98% male | 63 years | 57% White, 40% Black |
| 75 | Johnson, Catz, Remien, Rotheram-Borus, Morin, Charlebois, Gore-Felton, Goldsten, Wolfe, Lightfoot, Chesney & NIMH Healthy Living Project Team (2003) | N=2765 | Male (2054)  Female (674)  Transgender (35) | 17–29 (88) 67.05  30–39 (994) 65.90  40–49 (1235) 68.10  50–59 (382) 73.04  60–92 (59) 81.36 | Black/African American (1366)  Hispanic/Latino (504)  White (722)  Other (171) |
| 76 | Johnson, Chesney, Goldstein, Remien, Catz, Gore-Felton, Charlebois, Morin & NIMH Healthy Living Project Team (2006) | N=2765 | Male 74.3  Female 24.4  Transgender 1.3 | Age (years) 42 (7.6) | Black/African American 49.4  Hispanic/Latino 18.2  White 26.1  Other 6.2 |
| 77 | Johnson, Neilands,  Dilworth, Morin,  Remien & Chesney (2007) | Study 1 baseline N = 264  Study 2 baseline N = 2848  Study 2 follow up  N = 232 | Study 1 baseline:  227 male, 30 female, 7 other  Study 2 baseline:  2110 male, 699 female, 40 other  Study 2 follow up:  192 male, 40 female | Study 1 baseline 46.5 (8.1)  Study 2 baseline 42.5 (7.6)  Study 2 follow up  42.3 (7.5) | Study 1 baseline:  49 Black, 145 White,43 Latino, 27 Other  Study 2 baseline:  1375 Black, 754 White, 527 Latino, 190 Other  Study 2 follow up:  94 Black, 79 White, 39 Latino, 20 Other |
| 78 | Kalichman, Kalichman, Cherry, Swetzes, Amaral, White, Jones, Grebler & Eaton (2011) | N=40 | Group1: 13 men, 8 women, 1 transgender  Group2: 13 men, 6 women, 2 transgender | Group1:51.1 (sd 4.7)  Group2: 50.9 (sd 7.7) | Group1: 19 African American, 2 White  Group2: 18 African American, 1 White |
| 79 | Kalichman, Rompa, DiFonzo, Simpson, Austin, Luke & Buckles (2001) | N=112 | women | The median age was 38 years  (range = 24 to 62 years) | African American (n = 98, 88%),  10 (9%) White  4 (3%) being of other ethnic backgrounds |
| 80 | Kamau, Olson, Zipp & Clark (2011) | N=354 | female (71.47%) | Forty-two percent (42.2) of participants ranged from 31 to 40  years. | N.A. |
| 81 | Kamolz (2002) | N=182 | 107 male, 75 female | 51 years (range: 33-68) | N.A. |
| 82 | Katerndahl (2001) | N=72 | N.A. | N.A. | Anglo-Americans |
| 83 | Kaya, Erkan, Ozkan, Ozkan,Kocaman, Ertekin & Direk (2009) | N=63 | 50 female | 43 ± 10.48 years | N.A. |
| 84 | Kennedy, Goggin & Nollen (2004) | N=201 | 85% male, 14% female, 1% transgender, | 40 (from 18 to 66 years) | 55% Caucasian, 38% African  American, 5% Hispanic. Remaining participants were Native American  or Biracial. |
| 85 | Kerr, Marshall, Walsh, Palepu, Tyndall, Montaner & Wood (2005) | N=160  The mean age was | 91 (57%) were men and 69 (43%) were  women | 39 years (SD=7.04) | N.A. |
| 86 | Khdour , Hawwa, Kidney, Smyth & McElnay (2012) | 173 patients | 56 % females | mean age 67 | N.A. |
| 87 | Kim, Kim, Kim, Park, Chung & Chu (2011) | N=204 | 106 men, 98 women | 59.14 (SD= 11.64) | N.A. |
| 88 | Lamba, Nagurka, Desai, Chun, Holland & Koneru (2012) | N=237 | 152 male, 82 female | 20–49 years (n=42)  50–82 years (n=195) | African American 18  Asian/Asian Indian 12  Hispanic 35  Caucasian 169 |
| 89 | Lewis, Schoenthaler & Ogedegbe (2012) | N=253 | men | 56.6 (sd=11.6) years | Black |
| 90 | Li, Huang, Wang, Fennie, He & Williams (2011) | N=202 | 144 male, 58 female | 18-35 70  36-49 112  50 or above | Han nation (n=196)  Minorities (n=6) |
| 91 | Liu, Malin, Diamant,Thind & Maly (2012) | N=303 | females | 51 years | Latinas (49 %)  White (34%) |
| 92 | Luszczynska, Sarkar & Knoll (2007) | 104 participants | 63.5% women | M = 34.73 years (S.D. = 8.88) | N.A. |
| 93 | Lynam, Catley, Goggin., Rabinowitz, Gerkovich, Williams, Wright & MOTIV8 (2009) | N=189 | Male: 73% | N.A. | African American: 57%  Caucasian: 34% |
| 94 | Mann, Ponieman, Leventhal & Halm (2009) | N=151 | 68 female | Mean age (SD) 57 (11) | English native language (n=65)  Latino (n=58)  Black (n=34) |
| 95 | MacDonell, Jacques-Tiura, Naar, Fernandez & ATN 086/106  Protocol Team | N=956 | biologically male  (68.8%) | M=20.66  (SD=2.36). | 72 % Black,  20.4% mixed race/other  4.6% as Hispanic or Latino/a  White, 1.2% as Native American/Alaskan  Native  0.8% as Asian/Pacific Islander  23.6% Hispanic or  Latino/a |
| 96 | Marc, Testa, Walker, Robbins, Shafer, Anderson & Berkman (2007) | N=980 | 799 male  181 female | 37 years (s.d.=9.5) | Non-Hispanic White (456)  Non-Hispanic Black (339)  Hispanic (163)  Asian/Pacific Islander (17)  American Indian/Alaskan/unknown (5) |
| 97 | McDonald-Miszczak, Maki, & Gould (2000) | N=90 | 31 women, 59 men | 71.7 years (sd=7.44) | N.A. |
| 98 | McDonough, Boyd, Varvares & Maves (1996) | N=30 | 15 men, 15 women | 62.3 years (sd=12.4) | 21 White  9 African American |
| 99 | Mishali, Omer & Heymann (2011) | N=119 | 67 women | 57.45 years (SD = 10.91) | N.A. |
| 100 | Mo & Mak (2009) | N=102 | 87.3% male | 41.9 years (SD=10.04) | N.A. |
| 101 | Mohr, Boudewyn, Likosky, Levine & Goodkin (2001) | N=101 | female (78.2%) | 41.7 (SD = 9.7) | White (79.2%) |
| 102 | Molassiotis, Nahas-Lopez, Chung, Lam, Li & Lau (2002) | N=136 | men (92.5%) | 39.9 years (SD=9.4) | 120 Chinese  13 Caucasians  3 other Asian origin |
| 103 | Molassiotis, Morris & Trueman (2007) | N=38 | Male: 30  Female: 8 | 41.4 years (SD = 10.7) | White: 30  Black: 8 |
| 104 | Molloy, Randall, Wikman, Perkins-Porras, Messerli-Bürgy, & Steptoe (2012) | N=165  Group1:50  Group2:115 | Group1: 86 male  Group2: 86 male | Group1: 57.5 (sd=10.33)  Group2: 63.56 (sd=11.08) | Group1: White (European): 82%  Group2: White (European): 89% |
| 105 | Morasco et al. (2013) | N=80 | Group1: 29 male  Group2: 23 male  Group3: 21 male | Group1: 55.0 (5.7)  Group2: 56.1 (6.9)  Group3: 53.7 (6.2) | Group1: White: 20  Group2: White: 21  Group3: White: 19 |
| 106 | Murphy, Greenwell & Hoffman (2002) | N=46 | women | 37 years (sd=6.7) | 58.7% African American  23.9% Latina  6.5% White  10.9% mixed or other |
| 107 | Myers & Myers (1999) | N=31 | 3 females  18 males | 28.05 (SD=6.67) | N.A. |
| 108 | Naar-King, Templin, Wright, Frey, Parsons & Lam (2006) | N=24 | 38% male, 62% female | 21 years | 88% African American |
| 109 | Náfrádi, Galimberti, Nakamoto & Schulz (2016) | N=109 | 69 male | Group1:61.94 years (sd=9.78)  Group2: 61.94 (sd=13.29) | Caucasians |
| 110 | Nokes, Johnson et al. (2012) | N=1414 | Male 1,019 (71.1)  Female 372 (26.3)  Transgender 28 (2) | 46.6years (8.9) | Asian/Pacific Islander 61 (4.3)  African American/Black 567 (39.5)  Hispanic/Latino 348 (24.3)  Native American 28 (20)  White/Angelo 374 (26.1) |
| 111 | O’Hea, Moon, Grothe, Boudreaux, Bodenlos, Wallston & Brantley (2009) | N=109 | 28 male, 81 female | 52 years (sd=11.16) | 81 African American  28 White American |
| 112 | O'Hea, Grothe, Bodenlos, Boudreaux, White & Brantley (2005) | N=109 | 28 male, 81 female | 52 years (sd=11.16) | 81 African American  28 White American |
| 113 | Parsons, Rosof & Mustanski (2008) | N=275 | male (76.7%, n = 211) | 43.7 years (SD = 7.25) | 55.6% African American  23.7% Latino/a |
| 114 | Pepper, Carpenter & DeVellis (2012) | N=172 | 61 male, 111 female | 50.9 years (sd=12.3) | White 162  Other race 10 |
| 115 | Peyrot & Rubin (1994) | N=164 | 58% men | 47.4 years | 70% White |
| 116 | Pinheiro, de-Carvalho-Leite, Drachler & Silveira (2002) | N=195 | 119 male | 35 years | N.A. |
| 117 | Ponieman, Wisnivesky, Leventhal, Musumeci-Szabó & Halm (2009) | N=261 | 82% women | 48 years | 57% Hispanic  30% Black |
| 118 | Raiz, Kilty, Henry & Ferguson (1999) | N=309 | 47% female, 53% male | 50.3 years | Caucasian (88%)  Non-White (12%) |
| 119 | Reach, Michault, Bihan, Paulino, Cohen & Le Clésiau (2011) | N=90 | male (n = 54) and  female (n = 36) | 59 years (sd=4) | N.A. |
| 120 | Remien, Bastos, Tertor, Raxach, Pinto, Parker, Berkman & Hacker (2007) | N=200 | 71 male, 29 female | N.A | 52.5% Black/Mulatto  44.5% White  3% Other |
| 121 | Resnick, Wehren & Orwig (2003) | N=152 | 74% female | 85.7 years (SD=5.5) | 99% Caucasian |
| 122 | Robbins, D’Aquila, Morgello, Byrd, Remien, Mindt & Rivera (2012) | N=17 | 14 male | 46 years (SD=5.3) | Puerto Rican (17) |
| 123 | Russell, Cetingok, Hamburger, Owens, Thompson, Hathaway, Winsett, Conn, Madsen, Sitler & Wakefield (2010) | N=37 | Male (23)  Female (14) | 60.38 years (SD = 4.46) | Caucasian (19)  African American (17)  Other (1) |
| 124 | Safren, Otto, Worth, Salomon, Johnson, Mayer & Boswell (2001) | N=84 | Gay men 59 (70%) Bisexual men 2 (2%) Heterosexual men 13 (15%)  Men who refused to answer sexual orientation question 2 (2%)  Heterosexual women 8 (9%) | 40 years (SD=8.5) | African American 26 (31%)  Caucasian 37 (44%)  Hispanic/Latino 16 (19%)  Other 5 (6%) |
| 125 | Sajatovic, Ignacio, West, Cassidy, Safavi, Kilbourne & Blow (2009) | N=140 | Group1 (113): Male 57 (50.4)  Group 2 (27): Male 13 (48.2) ), Female 14 (51.8) | Group1 (113): 43.1 (sd=11.18)  Group 2 (27): 40.8 (sd=11.65) | Group1 (113): White 67(59.3), African American 18 (15.9), Other 28 (24.8) Group 2 (27): White 11 (40.7), African American 8 (29.6), Other 8 (29.6) |
| 126 | Sajatovic, Micula-Gondek, Tatsuoka & Bialko (2011) | N=140 | 70 men, 70 women | Group1 (113): 43.1 (sd=11.18)  Group 2 (27): 40.8 (sd=11.65) | Group1 (113): White 67(59.3), African American 18 (15.9), Other 28 (24.8) Group 2 (27): White 11 (40.7), African American 8 (29.6), Other 8 (29.6) |
| 127 | Samal et al. (2011) | N=433 | 286 (66%) male, 147 (34%) female | 45 years (0.45) | White 106 (24%)  African American 254 (58%)  Latino 62 (14%)  Other 15 (3%) |
| 128 | Sarkar,Fisher & Schillinger (2006) | N=408 | N.A. | 58.1 years (sd=11.4) | 18% Asian  25% African American  42% Latino/a  15% White |
| 129 | Scherer & Bruce (2001) | N=29 | 38% male (11) and 62%  female (18) | 53.71; SD, 14.6 | 90% White (26)  10% (3) Black |
| 130 | Schneider (1992) | N=137 | 54% male | 51 years | N.A. |
| 131 | Schneider, Wensing, Quinzler, Bieber & Szecsenyi (2007) | n=185 | Female 118 (63.8)  Male 67 (36.2) | 56.3 years (SD=15.9) | N.A. |
| 132 | Schoenthaler, Ogedegbe & Allegrante (2009) | N=167 | 85% female | 54 years | African Americans |
| 133 | Schousboe, Dowd, Davison & Kane (2010) | N=729 | Women: 681 (93.4%)  Men: 48 (6.6%) | 66.4 years (sd=10.1) | N.A. |
| 134 | Sevelius, Carrico & Johnson (2010) | N=2805 | Transwomen | 42 years (sd=7.6) | African-American (48.1%),  Caucasian (26.0%)  Hispanic/Latino (19.7%). |
| 135 | Shively, Smith, Bormann & Gifford (2002) | N=153 | Male 137  Female 16 | 41.41 years (SD=8.41) | White (not Hispanic) 96  African-American 31  Hispanic 19  Other 7 |
| 136 | Shon & Park (2002) | Group1 (N=18)  Group2 (N=20) | Group1: male: 10, female: 8  Group2: male: 12, female: 8 | Group1: 32,67 years (sd=8.13)  Group2: 32.95 years (sd=8.30) | N.A. |
| 137 | Simoni, Frick & Huang (2006) | N=136 | 61 (45%) women  ). | 42.6 years (SD= 8.9) | African American (46%)  Puerto Rican (39%) |
| 138 | Simoni, Frick, Lockhart & Liebovitz (2002) | N=50 | 31 women, 19 men | 41.45 years (SD=7.97) | African American (52%)  Puerto Rican (30%) |
| 139 | Stanton (1987) | N=50 | 56% male | 58 years | 47 White  2 Black  1 Hispanic |
| 140 | Theofilou (2013) | N=168 | 105 men (62.5 %), 63 women (37.5 %) | 62 years | Greek |
| 141 | Unni & Farris (2011) | N= 840  cholesterol lowering  medications: 420  asthma maintenance medications: 399 | cholesterol lowering medications:  males (54.35%)  asthma maintenance medications: female (61.4%) | cholesterol lowering medications:  59.4 years  asthma maintenance medications: 48.71 years | cholesterol lowering medications: White (85.5%)  asthma maintenance medications: 81% were white |
| 142 | Valeberg, Miaskowski, Hanestad, Bjordal, Moum & Rustøen (2008) | N=174 | men (n=37)  women (n=137) | 18-50 years: n=37  51-64 years: n=94  65-86 years: n=43 | N.A. |
| 143 | Voils, Steffens, Flint & Bosworth (2005) | N=84 | female (61%) | 67.67 years (SD=6.55) | N.A. |
| 144 | Wang, Bohn, Knight, Glynn, Mogun & Avorn (2002) | N=496  VAMC (n=248)  HMO (n=248) | VAMC  male (96%), female (4%)  HMO  male (38.7%), female (61.3%) | VAMC  below 55 (15.3%)  55-64 (23%)  65-74 (45.2%)  75+ (16.5%)  HMO  below 55 (21%)  55-64 (20.5%)  65-74 (35.5%)  75+ (23%) | VAMC  White (93.5%)  Non-White (6.5%)  HMO  White (96.8%)  Non-White (3.2%) |
| 145 | Warren-Findlow, Seymour & Huber (2012) | N=188 | 70% female | 53 years | N.A. |
| 146 | Warren-Findlow, Seymour & Shenk (2011) | N=190 | 71.60% female | 53.29 years (SD=16.12) | African American |
| 147 | Weng, Israni, Joffe, Hoy, Gaughan, Newman & Feldman (2005) | N=278 | 61.2% male | 48 years (median age) | 24.5% Black |
| 148 | Williams, Patrick, Niemiec,, Williams, Divine, Lafata, Heisler , Tunceli & Pladevall (2009) | N = 2973 | 45.3% female | 64.3 years (SD=10.5) | White (56.9%)  African American (38.4%)  Other (4.7%) |
| 149 | Williams, Rodin, Ryan, Grolnick & Deci (1998) | N=126 | 95 women, 31 men | 56.3 years (SD=7.52) | N.A. |
| 150 | Wilson, Doxanakis & Fairley (2004) | N=200 | 90% male | 45 years | N.A. |
| 151 | Wolf, Davis, Osborn, Skripkauskas, Bennett & Makoul (2007) | N=204 | 79.9% male | 40.1 years (SD=9.2) | 45.1% African-American |
| 152 | Wulandari,Craig & Whelan (2013) | N=299 | women | 26.3 years (SD=4.9) | Balinese (65.1%) |
| 153 | Yu, Yeoh, Seow, Luo & Griva (2012) | N=20 | Female: 8  Male: 12 | 64.4 years (SD=11.6) | Chinese: 12  Malay: 6  Indian: 2 |
| 154 | Zwibel, Pardo, Smith, Denney & Oleen-Burkey (2010) | N=146  Adherent (N = 126)  Non-adherent (N = 20) | Adherent:  Female (79.4%), Male 26 (20.6%)  Non-adherent:  Female (75.0%)  Male (25.0%) | Adherent: 43.2 years (SD=10.3)  Non-adherent: 42.5 years (SD=10.6) | Adherent:  Minority (15.1%)  Non-minority (69.9%)  Non-adherent  Minority (25%)  Non-minority (75%) |

N.A.: not available
